# A review of image processing and analysis of computed tomography images using deep learning methods

**DOI:** 10.1007/s13246-025-01635-w

**Published:** 2025-09-03

**Authors:** Darcie Anderson, Prabhakar Ramachandran, Jamie Trapp, Andrew Fielding

**Affiliations:** 1 School of Chemistry and Physics, Queensland University of Technology (QUT), Brisbane, QLD Australia; 2 Centre for Biomedical Technologies, Queensland University of Technology (QUT), Brisbane, QLD Australia; 3 Department of Radiation Oncology, Princess Alexandra Hospital, Brisbane, QLD Australia

**Keywords:** Radiotherapy, Computed tomography, Deep learning, Artificial neural networks

## Abstract

The use of machine learning has seen extraordinary growth since the development of deep learning techniques, notably the deep artificial neural network. Deep learning methodology excels in addressing complicated problems such as image classification, object detection, and natural language processing. A key feature of these networks is the capability to extract useful patterns from vast quantities of complex data, including images. As many branches of healthcare revolves around the generation, processing, and analysis of images, these techniques have become increasingly commonplace. This is especially true for radiotherapy, which relies on the use of anatomical and functional images from a range of imaging modalities, such as Computed Tomography (CT). The aim of this review is to provide an understanding of deep learning methodologies, including neural network types and structure, as well as linking these general concepts to medical CT image processing for radiotherapy. Specifically, it focusses on the stages of enhancement and analysis, incorporating image denoising, super-resolution, generation, registration, and segmentation, supported by examples of recent literature.

## Introduction

The discovery of radiation in the late nineteenth century quickly opened the way for radiotherapy and medical imaging to become tools useful in medicine for diagnosing and treating disease without invasive surgery. Today, medical imaging encompasses a range of techniques or modalities, including X-ray radiography, X-ray Computed Tomography (CT), Magnetic Resonance Imaging (MRI), Ultrasound (US), Positron Emission Tomography (PET), and Single Photon Emission Computed Tomography (SPECT). Each of these systems are used to obtain information of the internal structure and function of the body for clinical diagnosis.

**Fig. 1:**
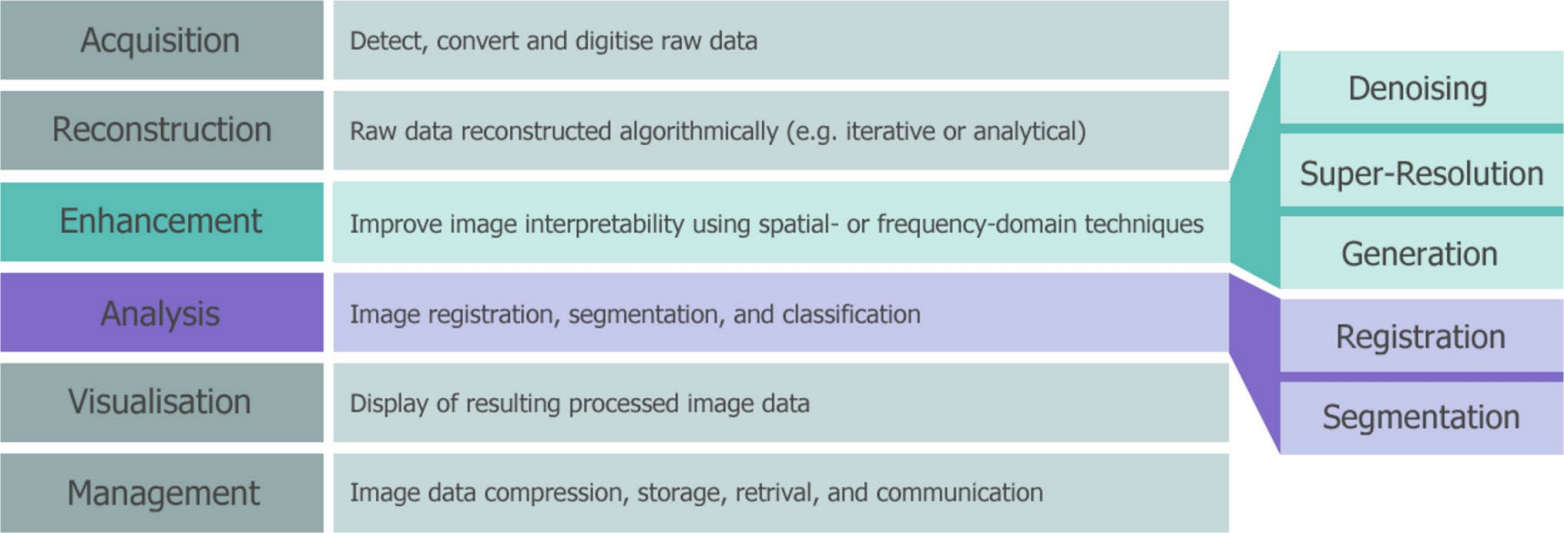
The six primary stages involved in medical image processing, with the pre-processing (enhancement) shown in green and processing (analysis) shown in purple. This review focusses on five derivatives of these two stages

Medical image processing is predominantly concerned with improving the interpretability of image features through six primary stages: acquisition, reconstruction, enhancement, analysis, visualisation, and management, as shown in Fig. 1. Following acquisition, the raw data may be reconstructed using either analytical or iterative mathematical algorithms before the image is interpreted by the clinical team. Image enhancement utilises techniques in the spatial or frequency domain to reduce noise, increase contrast, enhance edges, and minimise artifacts, among others. Enhancing the image quality assists with the subsequent analysis stage, which involves image segmentation, registration, and quantification. Segmentation focusses on dividing two-, three-, and four-dimensional reconstructions into regions of interest, which represent distinct anatomical or pathological structures, such as tumours or organs. Traditional computational segmentation methods are threshold-, edge-, and region-based [1]. Information from the delineated regions or volumes of interest is extracted through quantification, determining specific physical properties such as shape, size, texture, and composition. Image registration aims to align multiple images, either of mono- or multimodal origin, together into a shared coordinate system through geometric transformation, similarity measures, and optimisation algorithms. Although images may be visualised throughout the process, the visualisation stage is the final rendering of the image data post-processing. Finally, image management is concerned with storing, retrieving, and communicating the resultant image data [2, 3].

The advancement of medical imaging technologies has resulted in an exceptional amount of data, which is information dense, variable, and multi-modal. Medical images are handled differently to natural images in deep learning, as they often possess a larger spatial resolution (e.g., > 300 × 300 pixels), a singular channel dimension (greyscale), and lower variation in pixel intensity. Pixel intensity values are especially important in CT and other electron-density-based imaging, as they are directly related to anatomical tissue through the Hounsfield Unit (HU) scale. The scale assigns a radiodensity to the pixel intensities, which have been linearly transformed using tissue attenuation coefficients, such that water is typically 0 HU and air is -1000 HU [4]. All processing stages require expertise, time, and extensive effort from practitioners, especially in areas such as Adaptive Radiotherapy (ART) [5]. Deep learning methodology, a sub-section of artificial intelligence, has recently provided an opportunity for improving the five medical imaging processes highlighted in Fig. 1 [6].

### Artificial intelligence, machine learning, and deep learning

The broader concept of Artificial Intelligence (AI) emerged in the 1950s, with the goal of developing a computing system that acts with minimal human intervention or completely autonomously [7]. These systems are designed to perform specific tasks using intellectual traits such as reasoning, planning, learning, and perception.

**Fig. 2:**
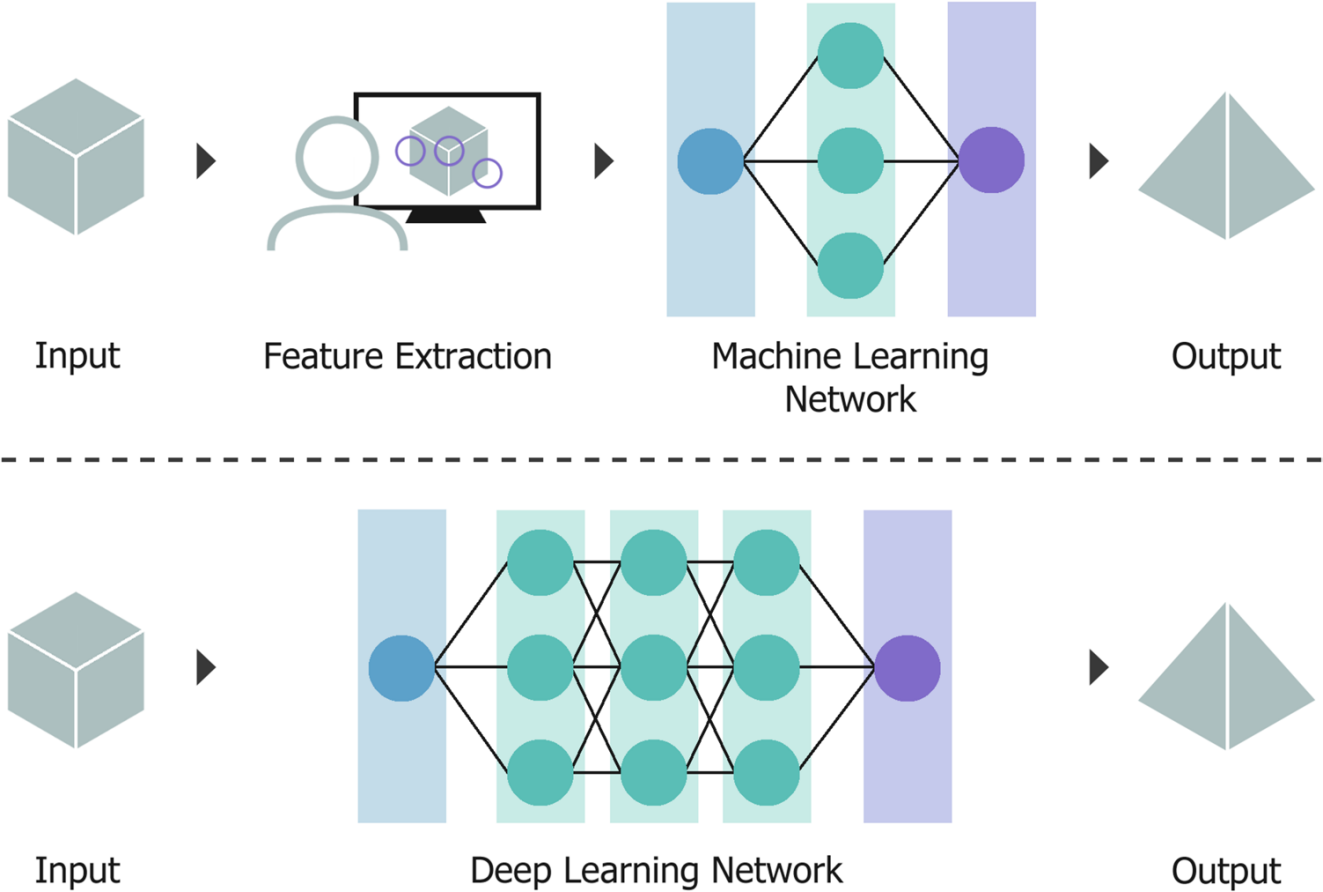
(Top): Simple machine learning workflow, with manual feature (characteristic) extraction and neural network design consisting of three layers: input (blue), hidden (green), and output (purple). (Bottom): Increasing the number of hidden layers between the input and output results in a deep learning neural network, allowing for automatic feature extraction. Each neuron in a layer is represented as a coloured circle

Within AI, Machine Learning (ML) was developed with the aim to provide computer systems with a method of “learning” to achieve the desired outcome from examples and observations, instead of traditional, more explicit programming. This reflects the human capability to improve performance through experience [8]. A machine learning algorithm is a multi-parameter predictive model fitted to a large, task-specific training dataset and validated on a smaller subset of data, before becoming capable of producing predictions on new or unseen data. The type of algorithm chosen depends on the problem at hand, with options including decision trees, regression models, Bayesian methods and Artificial Neural Networks (ANN) [9]. ANNs were initially inspired by the structure of the brain, a network of interconnected neurons that process inputs and are capable of learning from experience [10]. In machine learning, these neural networks have artificial neurons which are organised into layers; an input layer, zero or more “hidden” layers which have no direct connection to the environment, and an output layer. When the network is supplemented by more hidden layers, the depth of the network increases and a “deep” neural network is established [11]. A visual example of machine and deep learning neural network design is shown in Fig. 2.

Deep Learning (DL) is a sub-section of machine learning that excels at processing large datasets of high-dimensional data by using a combination of increased network layers, advanced neuron operations, and multiple activation functions [12]. Most machine learning algorithms, including simple ANNs, are “shallow” and require some form of human intervention [13]. However, in deep learning, the process of feature extraction, or identifying patterns in raw data, can be completed by the network during training [14]. The deep learning approach makes use of non-linear data processing for decision making by transforming data into higher, more abstract levels [15].

Overall, both styles are capable of tasks such as computer vision, prediction, and semantic analysis [16]. However, shallow machine learning algorithms are consistently outperformed by deep learning neural networks in situations involving large, high-dimensional data, such as speech, audio, images, and video [17]. The choice of using a shallow or deep learning algorithm, as well as the learning style, depends on the task and available data.

### Neural networks

Typically, deep learning neural networks are ANNs with several hidden layers situated between the input and output layers. In a neural network, each neuron receives input from connected neurons in the previous layer. It then computes the weighted sum of these inputs, considering the weights associated with each connection. In addition, the neuron adds a bias value to this weighted sum. Finally, an activation function is applied to determine the output of the neuron to introduce non-linearity to the network [11]. Using a hierarchy of layers, the goal of a neural network to transform non-linearly separable data inputs into linearly separable abstract features can be achieved [18]. As the data propagates forward through the network, characteristics (features) are learned with increasing complexity, from low- to high-level features. Low-level features are simple characteristics, such as lines, edges, and colours in an image, and individually have little relevance to the overall data. High-level features are formed from a combination of low-level features and represent more large-scale details like objects, scenes, and interactions [19].

The network learns to capture these characteristics through “experience” by training on large amounts of data. These large collections of data are known as “Big Data”, which describes the massive variety and amount of complex data that is continuously produced and stored, including data types that are unstructured and high-dimensional such as video, image, and text. Big Data is often defined by five characteristics: volume, variety, velocity, value, and veracity, known collectively as the 5 V’s. For a given dataset, the volume is the amount of data collected, variety is the diversity in the types of data present, velocity is the speed at which data is generated, value is the relevance or worth of the data, and veracity is the trustworthiness of the data. Professionals in medical imaging deal with this type of data every day, and deep learning neural networks are more capable of processing Big Data than shallow machine learning approaches [16, 17, 20].

Neurons within the hidden layers perform a linear mathematical operation on input data but require non-linear activation functions to learn abstract features from it. An activation function determines if the signal “strength” being received from a connection is sufficient for the neuron to be activated. Without a non-linear activation function, the network produces an output that is a linear function of the inputs, despite the network depth [18]. There are several types of activation functions, including sigmoid, SoftMax, hyperbolic tan (tanh) and Rectified Linear Unit (ReLU). The choice of activation function depends on the layer in which it is applied, a ReLU function is commonly used within hidden layers, whereas sigmoid and SoftMax are typically applied to output neurons in binary and multi-class classification tasks respectively [21].

The number of neurons is related to the “learning capacity” or robustness of the network, with too few neurons leading to inhibited learning (underfitting) and too many neurons leading to overlooking meaningful patterns (overfitting). Overfitting arises when the network becomes too specialised in one task without forming an understanding of the underlying connections, often failing to produce acceptable outputs when unseen data is introduced [22].

Mathematically, the output of the neural network, *y*, is calculated by applying an activation function, *f*, onto the sum of all the weighted input values, *x*, plus a bias value, *b*, as shown in Eq. (1) and (2).

1 $$y=f(\Sigma \left({x}_{i}\times {w}_{i}\right)+b)$$

2 $$\Sigma \left({x}_{i}\times {w}_{i}\right)= {x}_{1}{w}_{1}+{x}_{2}{w}_{2}+\dots +{x}_{n}{w}_{n}$$

**Fig. 3:**
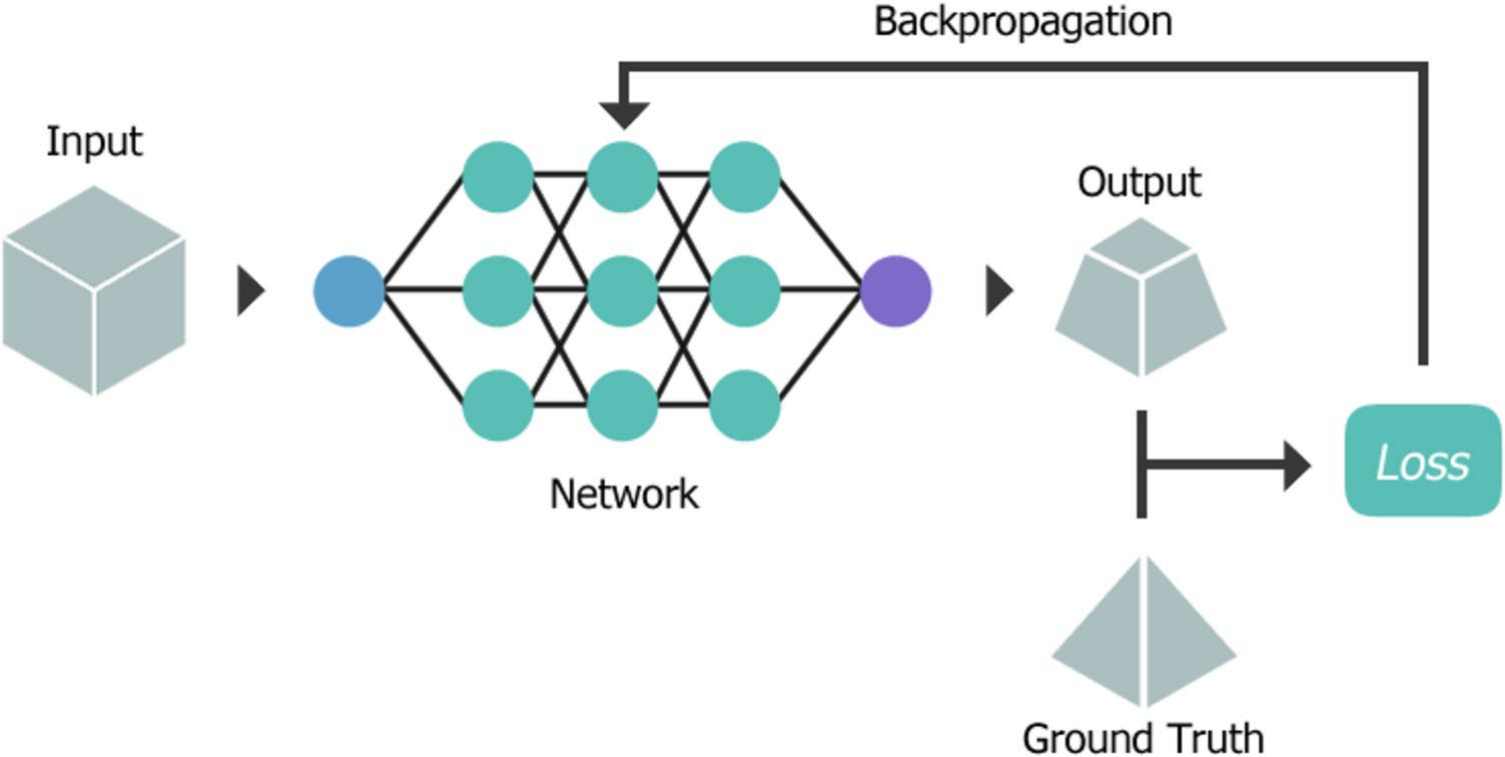
During training, backpropagation is utilised to update network parameters, such as weights and biases, to minimise loss between the network output and the ground truth or desired outcome

The goal of training a network is to repeatedly perform the chosen task (e.g., classification) on the data until the user-specified outcome is achieved through adjusting the set of weights and biases. After each iteration (epoch) of training, the capability of the network is evaluated by determining the “loss”, often by calculating the difference between the network output and the target or “ground truth” [23]. A “feedback loop” is created by propagating updated weights and biases backwards through the network, refining layer outputs and minimising the loss after each epoch in a process called “backpropagation”, shown in Fig. 3 [24]. Often, the input dataset is split into batches, which can improve the accuracy and stability of the network during training, as well as provide the opportunity to introduce randomness into the data, assisting in network generalisation [25]. Training continues until a stop condition is met, either after a maximum number of epochs or the loss value is sufficiently minimised. Following successful training of a well-generalised network, the network can then be tested using previously unseen data. Typically, the total dataset is split into unequal parts prior to training in either a train-test or train-test-validation split. The bulk of data is used for the training dataset, with the remaining portion used for testing and validation, however, the testing dataset is solely used to evaluate the network after training is completed. The validation dataset allows the network to tune hyperparameters during training, providing an unbiased evaluation [22].

#### Supervision

For researchers deciding which model suits the requirements of their deep-learning task, the choice is often data-dependent. Initially, a dataset consists of raw data that lacks identifying labels, which are included to convey information about its contents. The information given by the label is often derived from its purpose. There are two primary approaches for model training: unsupervised and supervised learning [26].

**Fig. 4:**
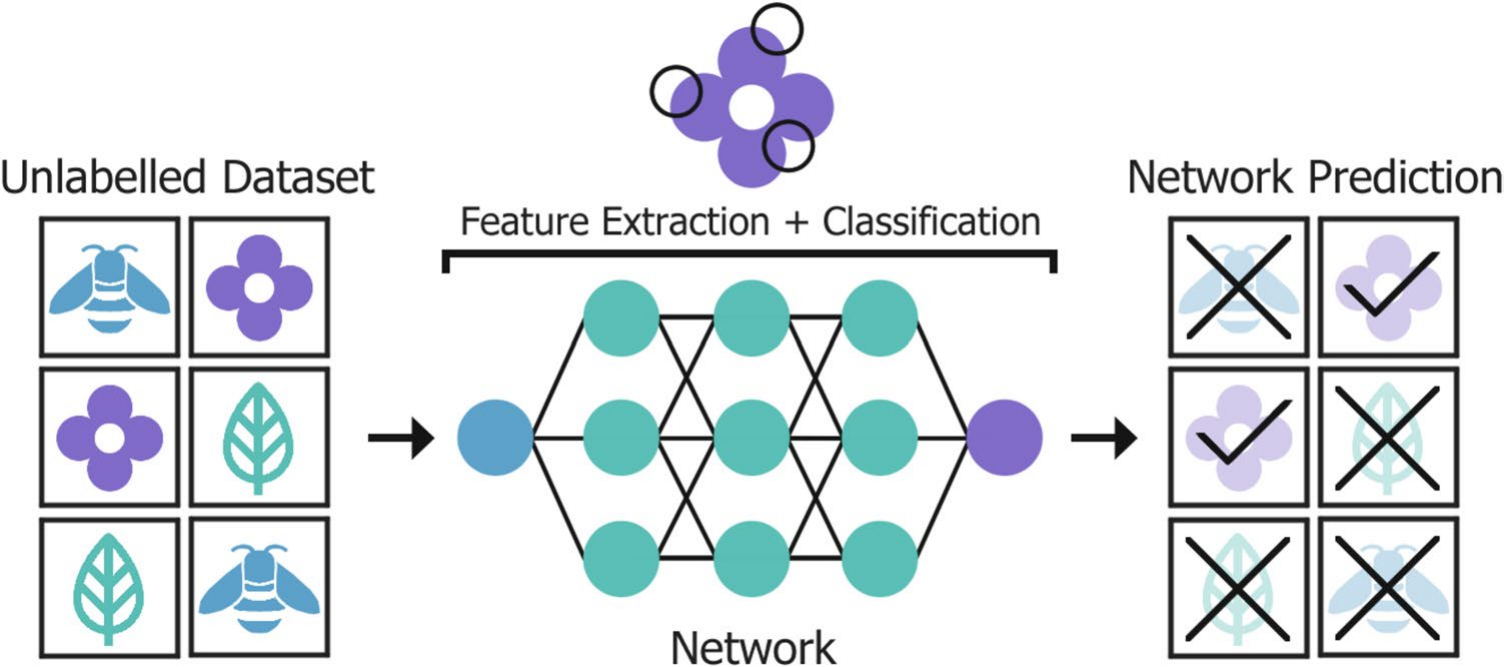
An unsupervised deep learning neural network is trained on an unlabelled dataset, learning to identify features automatically and classify the input images without guidance. Subsequent network testing follows a similar procedure

Unsupervised networks, like the one shown in Fig. 4, are trained to learn characteristics, patterns, and relationships from the raw input dataset directly and automatically, without labels or intervention. Alternatively, supervision provides guidance to the network, where the network is trained using a dataset of pre-labelled data. Prior to input, the data is paired with the appropriate corresponding label, creating examples or a target for the network to aim towards. This, however, can be a significant disadvantage because most available data is raw, and in most cases a large amount of accurately labelled data is required to adequately train a network. Generation of large, labelled datasets is very labour intensive, requiring manual labelling often by a skilled professional in the field [27]. Annotation is the process of labelling medical imaging data, conventionally carried out manually by a physician following a protocol. Common annotations for medical images include category, bounding boxes, masks, and tags. The contents of a CT image may be broken down into a single label which describes the contents more generally (e.g., acquisition type, imaging region, lesion type etc.), or more specifically within the image or volume through segmentations, regions of interest (ROIs), and landmarks [28].

**Fig. 5:**
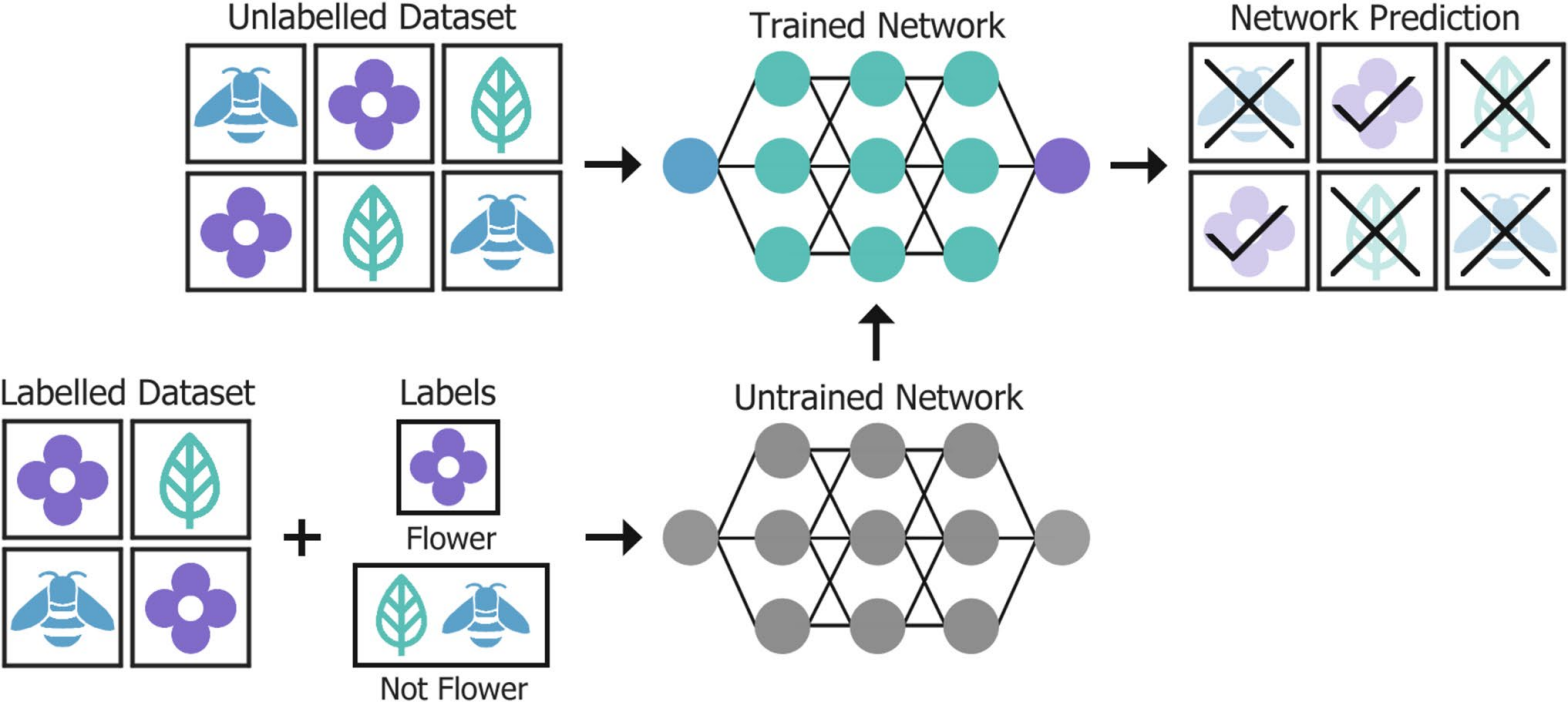
For supervised training the untrained network takes a labelled dataset and corresponding labels as input before testing. An unlabelled dataset is utilised to test the capability of the trained network to produce accurate predictions on unseen data

For example, a neural network may be given the classification task of distinguishing if an image depicts a flower by responding “yes” or “no”. The idea is to avoid explicitly programming a definitive list of defined characteristics. In supervised learning, as shown in Fig. 5, the network is given image input data with the corresponding binary classification label (yes or no) during training. It learns to correctly label the images by repeatedly observing flowers and using those observed characteristics to “guess” the correct label. However, an unsupervised network is not capable of achieving this, as there is no label to compare its prediction to. Primarily, an unsupervised network identifies patterns within the dataset and groups together data based on similarity, suitable for tasks such as feature extraction, anomaly detection, and data clustering. The most significant advantage of unsupervised learning is the reduction in labour due to the absence of data labelling, although methods for AI-assisted dataset labelling are in development [29, 30]. After sufficient training, the network should be able to identify if a previously unseen image is a flower or not, in a manner not dissimilar to humans [31].

### Building a neural network

There are several types of neural network configurations that can overcome the limitations of standard machine learning algorithms, such as needing to process large and high-dimensional data [17]. There are two primary approaches to neural network structure: feed-forward and feedback [32]. The former includes networks such as the perceptron, multi-layer perceptron, radial basis function networks, and convolutional neural networks, while many feedback networks arise from the structure of recurrent neural networks.

**Fig. 6:**
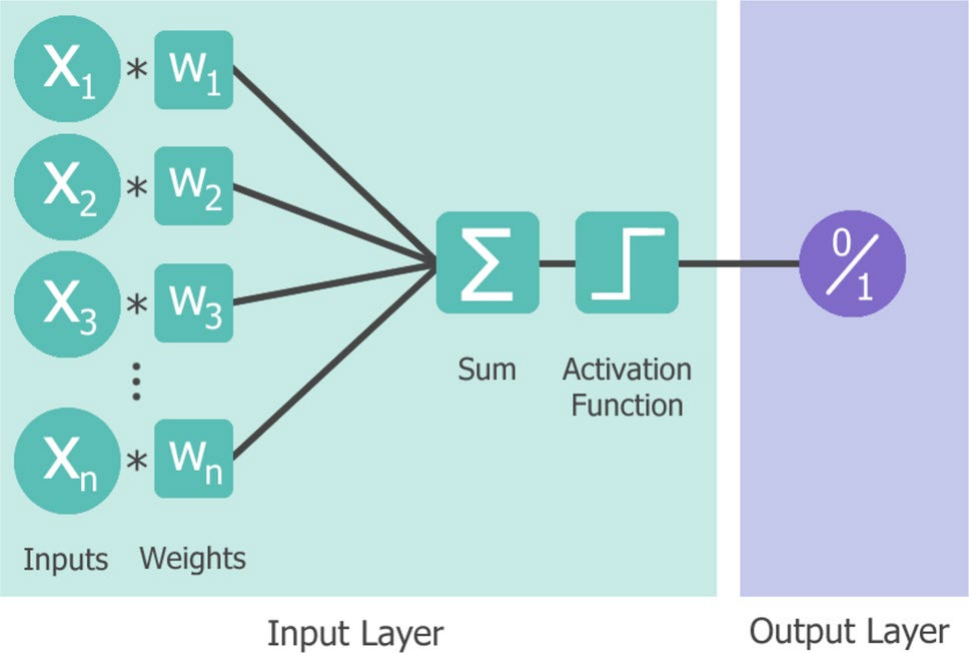
Perceptron network with x_n_ binary inputs, multiplied by weights before summation and activation, producing a binary output for classification

The perceptron is a neural network in the simplest form, as it possesses no hidden layers, and is capable of performing binary classification [33]. It only contains an input layer of one or more inputs, and single neuron as the output layer, as depicted in Fig. 6. Weighted input values are summed by the single neuron before being fed through the activation function to produce the binary output. This is the fundamental process that more complex neural networks are built from [34].

**Fig. 7:**
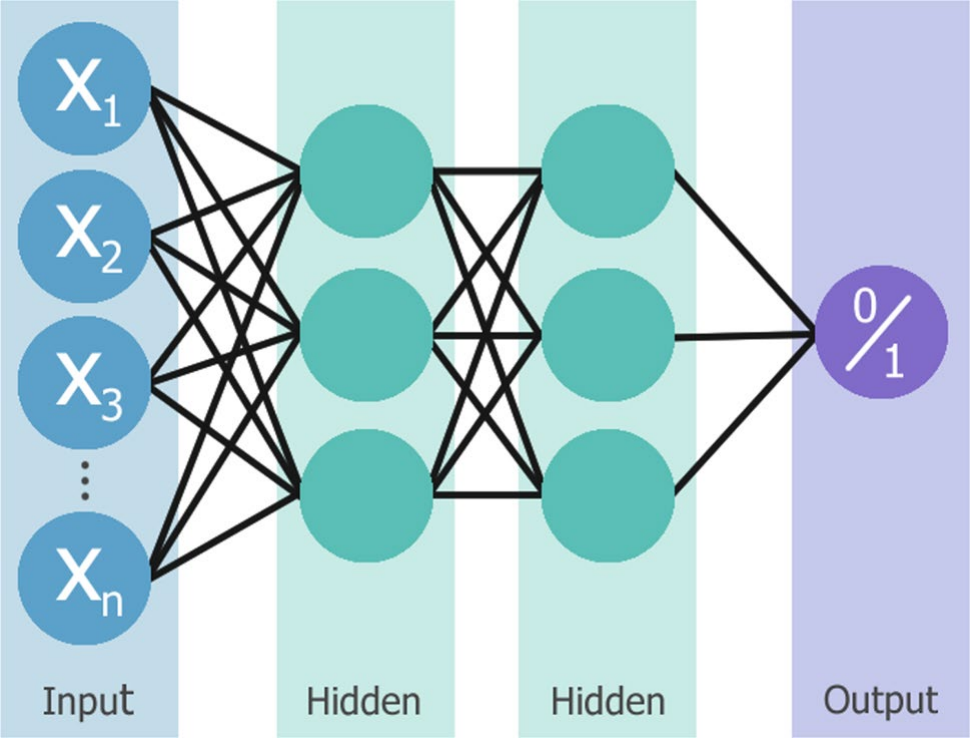
A multilayer perceptron network with two hidden layers positioned between the input and output layers, x_n_ inputs and a binary output

Additional layers can be added to the perceptron to make a multi-layered perceptron, a type of “fully-connected” neural network which is shown in Fig. 7. Slightly more complex, a fully-connected network is where each neuron is connected to every neuron in both the previous and following layers, without any connections between neurons in the same layer. Hyperparameters, such as the number of hidden layers present and the number of neurons within each layer (layer width), are specified by the user. Usually multi-layered perceptron networks utilise backpropagation, where the input is propagated forward and the weight updates are propagated backwards [35].

**Fig. 8:**
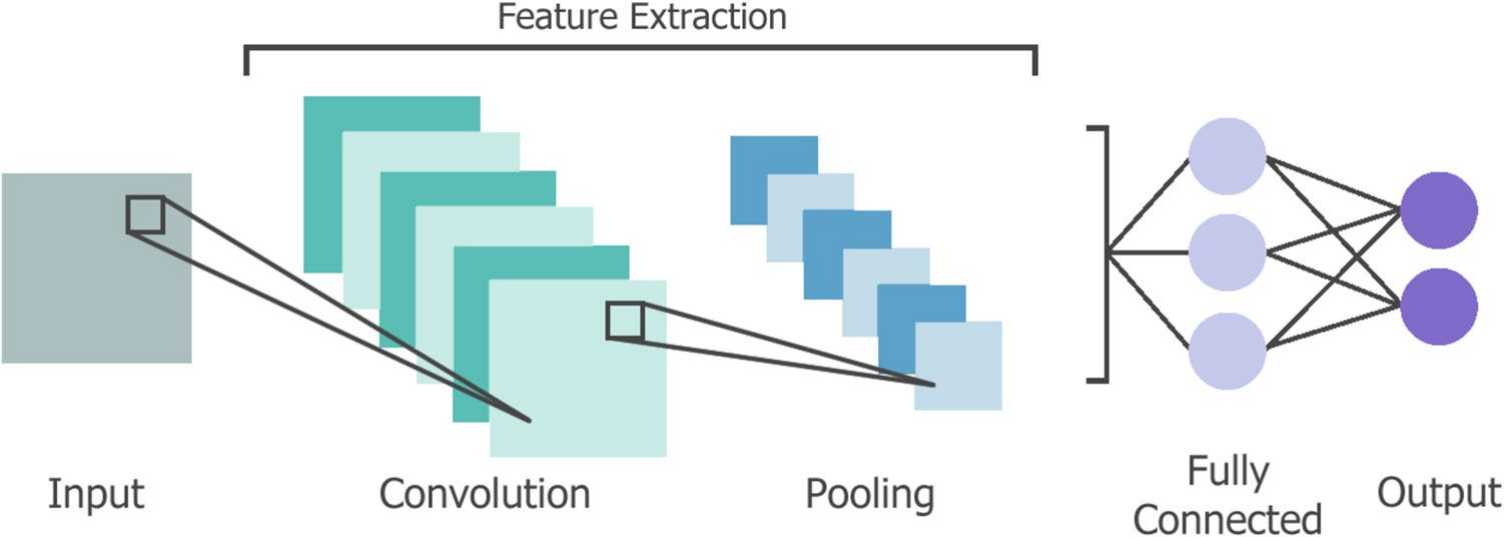
A simple convolutional neural network that extracts features from the input image using a single convolution-pooling block before a fully connected (FC) layer and the network output layer

Another type of deep learning architecture is the Convolutional Neural Network (CNN). Transformation neurons within hidden layers perform convolutions on the data, an operation which excels at image-based tasks such as classification, image recognition and object detection [36]. In its most basic form, a convolutional neural network consists of an input layer, a convolution layer with an activation function, a pooling layer, a fully-connected layer, and an output layer, as shown in Fig. 8 [37]. The convolution layer is responsible for identifying characteristics in pixels using a kernel, the pooling layer makes these features more abstract by downsampling, and the fully-connected layer uses the detected features for prediction. Each neuron in the CNN focuses on a region of the input image, known as the receptive field, determined by the convolution kernel size and stride. In this manner, only the contents of the receptive field influence the output of a neuron. Typically, as CNNs are hierarchical, neurons in the initial layers possess small receptive fields that learn low-level features, like edges and colours. As the depth through the network increases, so does the receptive field. A larger receptive field captures more information, learning high-level features, like objects and scenes [38]. Commonly, ReLU activations are used as the activation function in CNNs, and the “convolution-ReLU-pooling” block can be repeated to support the feature hierarchy introduced by the receptive field. During training, weights and kernels are updated via propagating backwards through the network layers [39].

#### Neural networks expanded: U-Net

**Fig. 9:**
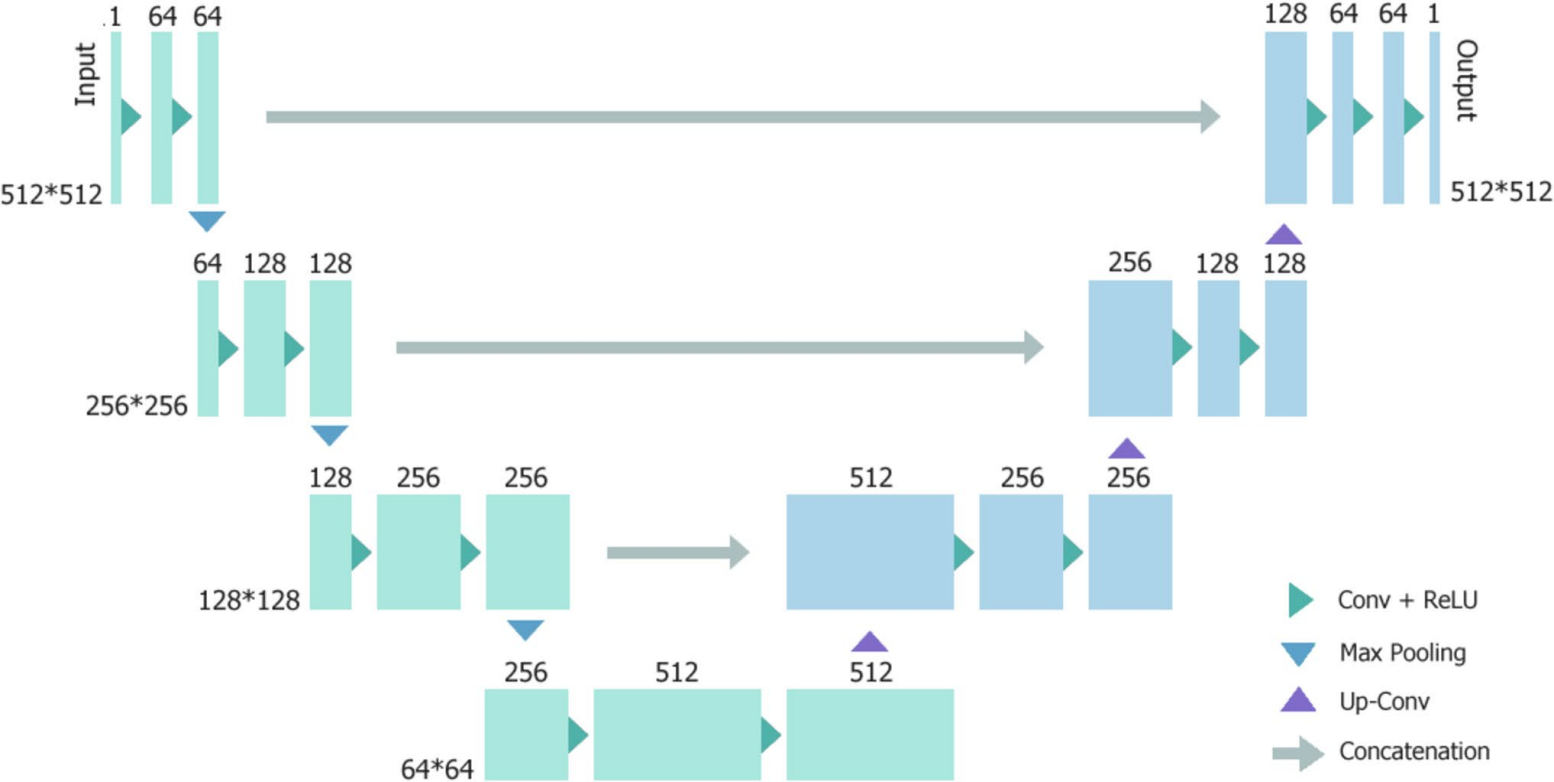
U-Net network architecture displaying the characteristic U-shape. There is a contracting path of three downsampling blocks (left) and an expanding path of three upsampling blocks (right), with the bottleneck in the centre. The spatial dimensions of the input image matrix are shown to the adjacent to each block, while the matrix depth is shown above

First introduced in 2015 by Ronneberger et al. [40] for biomedical image segmentation of cellular microscopy images, the U-Net is an CNN design comprised of a contracting path of encoder layers, followed by a mirrored expanding path of decoder layers. The contracting path utilises “convolution-ReLU-pooling” blocks to downsample the input, extracting contextual feature information, decreasing the spatial dimensions, and increasing the depth (feature channels). Growth in the number of channels allows for increasingly high-level features to be captured as the data moves forward through these encoding layers. The bottleneck following the encoder layers condenses and finalises the feature map, ensuring that it only contains useful information for the decoding path [41]. Each layer of the expanding path up-samples the feature map through transposed (or up-) convolutions instead of pooling, increasing the spatial dimensions and decreasing the feature channels. An example of a U-Net network with a depth of 5 is shown in Fig. 9, here network depth describes the number of blocks present in either path of the U-Net. This process transforms the data to the size of the original input and allows the network to localise the features. A unique characteristic of the U-Net is the inclusion of skip connections between the corresponding layers of the contracting and expanding paths. These connections concatenate the two feature maps together (contracting and expanding) to assist in preserving spatial information that is lost in the encoder layers. The output of the network is a segmentation map consisting of two or more classes, with each pixel of the map being assigned a label corresponding to a class in the input image. In a CT image, individual pixels may be given labels corresponding to the tissue type (class), such as “organ”, “bone”, or “tumour”. The U-Net is a hugely successful network that has applications in a variety of medical imaging tasks, including treatment area segmentation, imaging modality transfer, and synthetically generated image creation [42].

#### Neural networks expanded: generative adversarial network

**Fig. 10:**
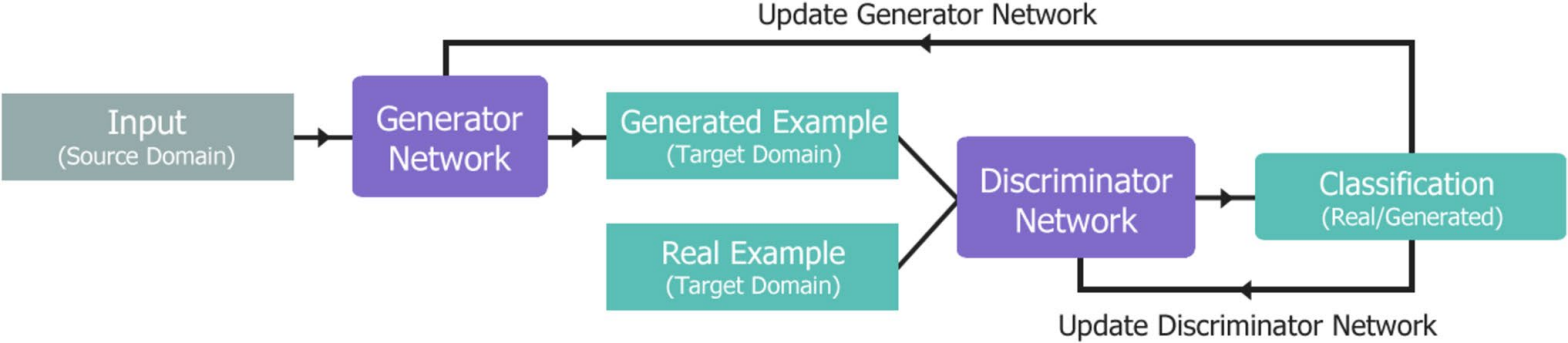
Generative Adversarial Network (GAN) model structure. Input from the source domain is used to generate examples in the target domain. The discriminator network takes a generated example and a real example from the target domain, and classifies both as either real or generated. Parameters for both networks are then updated

**Fig. 11:**
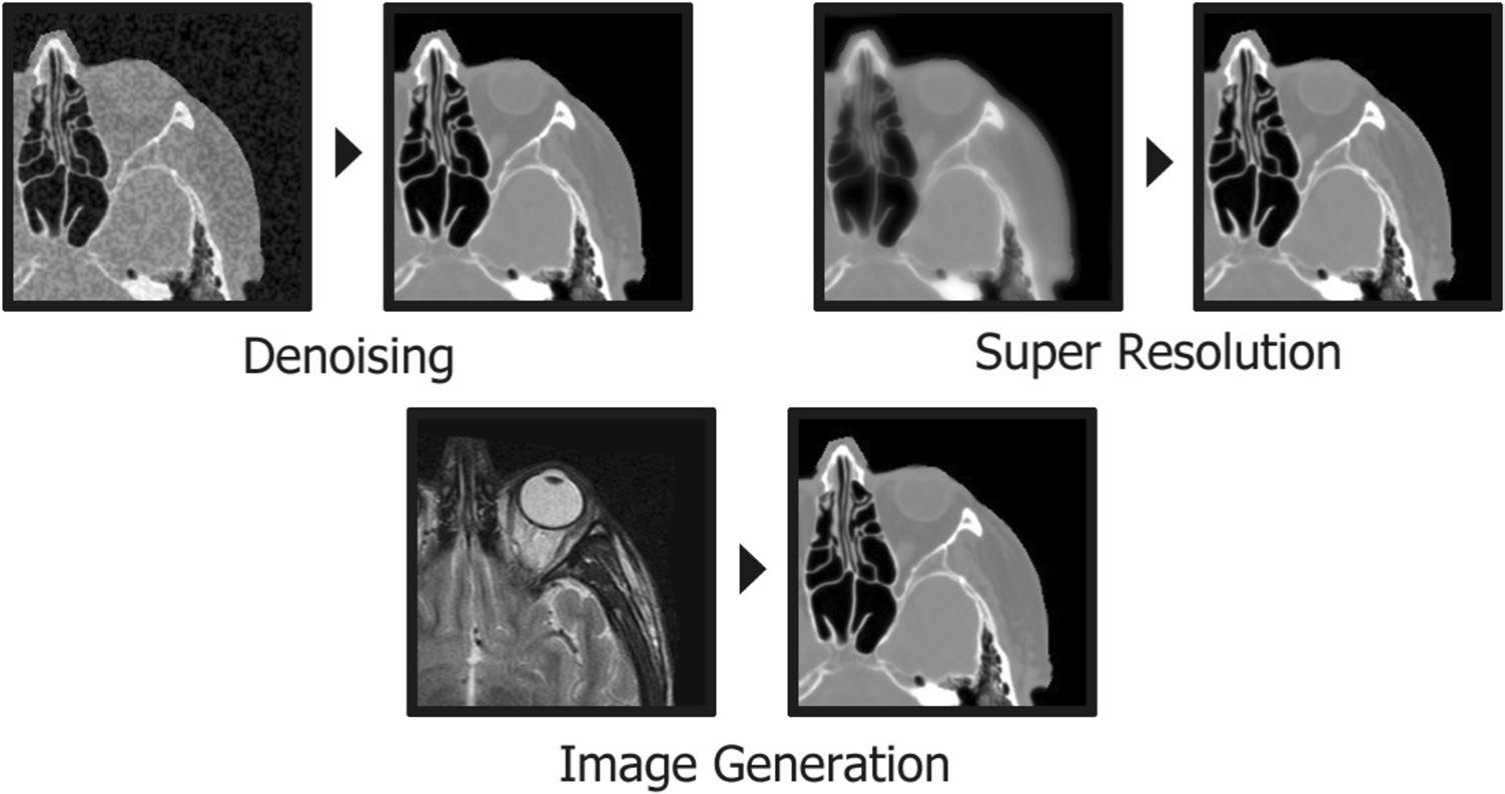
Image enhancement techniques employed to improve image quality. Denoising reduces the noise present in an image, super resolution decreases blur and increases resolution, and image generation creates a synthetic image from the input

Generative modelling aims to discover and learn features in given input data for the purpose of generating new outputs that are almost indistinguishable from the original dataset. This is especially useful for approaching the issue of data scarcity in medical imaging, through generating high-quality samples which are incorporated into the large datasets required to train neural networks [43]. Goodfellow et al. [44] proposed the Generative Adversarial Network (GAN), shown in Fig. 10, to address generative modelling by incorporating two networks that are trained competitively: a generator and a discriminator. The generator network aims to synthesise new outputs by learning to map an input from the source domain to the target domain. The discriminator network is given a batch of samples from both original data and the generated outputs that it must classify as either real or fake. If all of the samples are successfully identified by the discriminator, then the generator is heavily penalised using substantial parameter updates. The inverse is true if the generator fools the discriminator successfully. Although unnecessary, ideal training converges on the discriminator being “unsure”, predicting a 50% chance of being real, 50% fake in every given sample. CNNs are utilised as the basis for both networks, such as a U-Net generator and PatchGAN discriminator in the Pix2Pix image-to-image translation GAN model proposed by Isola et al. [45] They developed a conditional GAN (cGAN), which generates samples from a given domain type by incorporating labels in the training process for guidance (conditioning). For the generator, a specific label directs the type of output generated, such as a rose instead of “flower” (Fig. 11). Instead of a traditional GAN, Pix2Pix utilises a PatchGAN discriminator design, which classifies smaller regions or “patches” of the whole image, improving the quality of generated images [46]. GANs are a very successful and varied CNN design, finding applications in the generation of images, video, and audio, as well as image-to-image translation, object detection and segmentation [47]. They have been utilised in medical imaging for tasks such as synthetic CT image generation from MRI data, super-resolution of low-dose CT images, and contouring treatment areas.[48].

#### Neural networks expanded: vision transformer

Generational tasks do not have to be handled by convolution-based networks, alternatively, convolution operations can be done away with in favour of self-attention. Derived from its natural language processing counterpart, the Vision Transformer (ViT) was introduced by Dosovitskiy et al.[49] in 2020 to compete against the dominance of convolution-based neural networks through a mechanism called “self-attention”. Similar to CNNs, transformer architecture is generally comprised of stacked layers, namely: a tokeniser module, an embedding layer, the transformer blocks, and an unembedding layer. Conventional transformer models deal with textual inputs by converting them to tokens via the tokeniser, which numerically represent a single character or a short sequence of characters (i.e., a whole word or part thereof). These tokens are then transformed into vector representations by multiplying the one-hot token representation with an embedding matrix. To account for the order of the input sequence, each token also has a positional encoding, which is the vector representation of its relative position within the sequence. The two vectors, the embedding vector from the token and the positional encoding, are combined before they are processed by the transformer layers through attention [50]. In a ViT, the input is instead an image that has been divided into a sequence of flattened two-dimensional patches that are tokenised through a linear projection of the patch. After tokenisation and embedding, the transformer blocks utilise self-attention to weigh the relative importance of each token in the sequence. Each block has an “attention head” that calculates an attention score based on three variables: query (Q), key (k), and value (V). The query vector represents the current focus of the sequence, the key vectors represent the other vectors in the sequence, and the value vector determines its contribution to the final output. The encoded vectors are transformed into the three vectors via multiplication with the corresponding matrices (W(Q), W(K), and W(V), respectively). The attention score is calculated via the dot product of the current query and each key, indicating the relevance of each key to the query, before they are combined with the value vectors. The final output for a single query of an attention head is the weighted sum of the value vectors. In addition, the attention mechanism can be extended to multi-head self-attention, which incorporates several self-attention blocks which process the layer input in parallel before concatenating the output. Despite the advantages over CNN-based models, the use of transformers is generally limited by long training times, model complexity (number of parameters), and the requirement for large training datasets for adequate generalisation [51].

### Applications of neural networks in medical imaging

Deep learning finds uses in analysing and extracting useful information from large data quantities in a wide variety of applications familiar to machine learning, such as computer vision, prediction, and semantic analysis, natural language processing, and information retrieval [52]. The medical field has benefitted greatly from the introduction of deep learning methodology in areas such as clinical decision support [53], predictive analysis [54], drug discovery [55], genomics [56], and monitoring [57]. Recently, a trend has grown to incorporate the successes of deep learning in image processing into medical imaging through image classification, segmentation, registration, and generation [58]. A variety of neural networks have been assessed in literature, with a particular interest in using CNNs and other deep learning architectures [59]. This review will focus on the deep learning techniques utilised in current literature and developments regarding medical image processing in diagnostic radiology, specifically in Computed Tomography (CT) with a focus on areas of image enhancement and analysis. Examples of recent, complementary reviews that discuss the applications of deep learning applications in medical imaging are outlined here.

#### Classification

Traditionally, physicians manually perform medical image classification to identify and diagnose disease using collected imaging data by analysing the contents of the image. However, this process is time-consuming and carries a degree of variation due to human input. In deep learning, the aim is to train a neural network to automatically extract features from an input image or volume and subsequently assign a predefined category informed by those features. Dao et al.[60] comprehensively review several deep learning strategies utilised in recent research for the purpose of medical image classification, with a focus on interpretability through eXplainable Artificial Intelligence (XAI). As reliability, accuracy and data security are critical in the medical field, the conventional “black box” interpretation of deep learning methods has led to some resistance in adoption in clinical settings. XAI attempts to clarify the internal processes and reveal the logic supporting the decisions of the network [61]. As their attention is on the learnable task (i.e., classification), they examine many applications of neural network architectures broadly, covering several imaging modalities throughout the review. Consequently, the characteristics of a specific imaging modality and their impact on deep learning approach is less comprehensive. Additionally, only CNNs, ViTs, and Vision-Language Models (VLMs) are discussed, the emerging development of diffusion-based models and other techniques are not investigated.

#### Segmentation

An integral part of the workflow in the analysis of medical images is the segmentation or division of anatomical structures into distinct regions of interest (ROIs) for diagnosis, treatment planning and monitoring. Typically, segmentation is achieved through manual contouring or algorithmically through methods including thresholding [62], clustering [63], region-based [64], and edge-based [65]. Semi- or fully-automated segmentation workflows decrease time and labour required for manually delineating anatomical structures, as well as assist in reducing inter- and intra-observer variability [66]. A recent review published by Xu et al. [67] explores the effectiveness of different approaches to segmentation, including traditional and deep learning-based techniques. They present examples of literature featuring several well-known deep learning architectures (e.g., CNNs, GANs, fully-convolutional networks (FCNs), recurrent neural networks (RNNs), and autoencoders (AEs), reporting the accuracy of each approach in delineation tasks. Despite the comprehensive examination of these neural networks, the review lacks coverage of other emerging architectures, such as transformers. Transformers overcome the sequential nature of RNNs, which cannot parallelly process all tokens in a sequence, and have seen success in computer vision tasks like medical image segmentation [68]. In their discussion of applications of deep learning in medical image segmentation, the authors categorise each approach by anatomical region, covering a number of studies concerning the brain, breast, liver, lung, prostate, retina, and skin.

#### Registration

Conventionally, rigid and non-rigid medical image registration is carried out algorithmically through iterative optimisation. Registration techniques such as Elastix [69], demons-based [70], and large deformation diffeomorphic metric mapping (LDDMM) [71] are highly interpretable and explainable, allowing physicians to understand the internal processes of the algorithm. However, these approaches focus on iteratively optimising a distinct transformation for each pair of images, which can be slow and computationally intensive, especially if volumetric multimodal imaging is involved [72]. The goal of deep learning methods is to learn a global registration function, shortening registration time while maintaining accuracy [73]. Chen et al.[74] survey several neural network architectures and their contribution to registration in single- and multi-modal instances, noting the similarity to other computer vision tasks in the dominance of CNNs. Out of the sixty-four methods outlined, approximately 70% were reported as utilising a CNN or CNN-derived architecture. Despite this, alternative designs like Transformers and diffusion-based networks are comprehensive explored, with examples of recent publications included. As the focus of their review is on the loss function selection and estimation of registration uncertainty, similarity measures are only briefly covered. Similarity measures are utilised extensively in medical image registration to quantify the agreement between the two images, determined through techniques such as cross-correlation and mutual information [75].

#### Generation and translation

Generally, image generation refers to the synthesis of an image from an abstract (unconditional) or specific (conditional) input. Conditional generation or translation has a strong relationship between the source and target domains (i.e., the “styles” of the input image and desired image), such that the size and location of structures are preserved. As the specific content of a medical image is crucial to its clinical relevancy, image generation in medical imaging commonly revolves around translation tasks, that is, converting one medical image to the style of another. Primarily, translation eliminates the need for multi-modal image acquisition for one patient [76]. Both intra- and inter-modal image translation tasks (e.g., low-dose CT to high-dose CT, and MRI-to-CT, respectively) are reviewed by Chen et al.[77]. A variety of neural network architectures are covered in the review, including widely-used networks like GANs, variational auto-encoders (VAEs), autoregressive models (ARs), transformers, diffusion models, and flow models. Although cone-beam CT (CBCT) is discussed with respect to common datasets utilised in medical image translation tasks, examples of publications focussing on CBCT data are not included within their scope. They outline the open-source SynthRAD 2023 dataset [78], which was developed for synthetic CT image translation from two sources: MRI (for MRI-CT translation) and CBCT (for CBCT-CT translation). Integrated kilovoltage CBCT units are an effective tool in image-guided radiotherapy (IGRT), frequently utilised for patient position and treatment verification, motion tracking, and interfractional corrections to treatment plans [79].

### Statement of purpose

This work aims to provide a foundational understanding of deep learning methodologies, as well as highlight examples of advancement in its application in CT for radiotherapy. It sets out to give medical professionals who are interested in deep learning a summary of the different models currently in use by researchers in their field, as well as the problems to which they address. It focusses on five key areas where deep learning methodologies has been applied to CT imaging, outlining recent publications in each area. These areas are image denoising, super resolution, generation, registration, and segmentation. The database Scopus was utilised to collect the relevant research.

Studies outlined herein are chosen as emergent examples of parallel development in deep learning approaches which focus on the same goal or task: CT image enhancement and analysis. They are limited to quality works published in English within the last three years, spanning from the beginning of 2022 to 2024. Older works have been previously covered in reviews such as Jung et al. [80] Publications which introduce novel deep learning models alone or a complete end-to-end workflow are included. No works were excluded due to the cohort of the study, such as number of subjects or imaging location (e.g. head-and-neck, abdominal). However, reviews, conference papers etc., are excluded from the criteria, as they are not the original or primary sources. For the review of current literature, search terms corresponding to the area of deep learning application were used, which are as follows:

i) Image denoising: “deep learning denoising computed tomography”
ii) Image super resolution: “deep learning super resolution computed tomography”
iii) Image generation: “deep learning generation computed tomography”
iv) Image registration: “deep learning registration computed tomography”
v) Image segmentation: "deep learning segmentation computed tomography”

The terms “deep learning” and “computed tomography” were shared amongst all search criteria to ensure that the results are relevant to this review.

In summary, the aim of this work is threefold:

1. Explore the core properties of deep learning methodology, explaining the basic components of neural network architecture.
2. Highlight examples of advancement in the application of deep learning, namely neural networks, through presentation of recent published literature in enhancing and analysing medical CT images.
3. Identify challenges faced in applying deep learning to medical imaging.

## Literature review – medical image processing in computed tomography (CT)

The successes of deep learning methods in non-clinical image processing have led researchers to evaluate them in a variety of medical imaging modalities, including CT. CT provides a visualisation of the internal structure of the body at the tissue level by using an x-ray beam and detector array that rotate around the patient. X-ray photons in the beam are attenuated by differing amounts as it propagates through the tissues within the imaged patient. The resulting reconstructed imaging dataset is a 3D x-ray attenuation map of the different tissues within the imaged patient. As these various tissues possess distinct attenuation coefficients, they can be differentiated in the final reconstructed image, where high-density materials such as bone or metal are bright and low-density materials are dark. The beam is rotated about the subject, creating a series of cross-sectional slices that are used to reconstruct a 3D volumetric image [81]. Cone-beam Computed Tomography (CBCT) is based on the principles of CT but utilises a kilovoltage conical x-ray beam and a flat panel detector to capture the data for an entire volume with a single rotation [82]. CBCT is often incorporated as an auxiliary system to assist in pre- and on-treatment radiotherapy patient positioning, known generally as Image-Guided Radiotherapy (IGRT), as well as providing the potential to modify or adapt treatment plans to changes in the patient over the course of a treatment, known as Adaptive Radiotherapy (ART) [83]. Due to the beam characteristics and cone-shaped geometry, issues such as noise and artifacts common to conventional CT are often more severe in CBCT [84].

Although a variety of anatomic and functional imaging modalities exist, such as Magnetic Resonance Imaging (MRI), Positron Emission Tomography (PET), Single Photon Emission CT (SPECT), and Ultrasound (US), CT-based imaging remains the standard for a range of applications [85, 86]. Imaging in radiotherapy is predominantly centred around CT, as it provides material density information that can be used for dose calculations, while the same isn’t true for other modalities like MRI. For example, MRI provides better soft-tissue contrast, but is limited by factors such as high cost, availability, long scan times and the increased impact of motion. CT can also combined with other techniques for multimodal imaging, such as PET-CT [87] and SPECT-CT [88], enabling more precise anatomical localisation, combined with functional information provided by PET and SPECT.

### Enhancement and analysis stages of medical imaging

#### Image quality

Image quality in medical imaging plays a crucial role in the accurate diagnosis and planning of therapeutic interventions, where achieving clear delineation of anatomical and pathological structures is essential. Additionally, minimising image noise and artefacts is vital to ensure informative and reliable diagnostic images. Several factors influence the quality of an image: contrast, spatial resolution, image noise, and artefacts.[89] In Table 1, some common metrics used to quantify image quality are briefly described. The Dice Similarity Coefficient (DSC), Hausdorff Distance (HD), and Target Registration Error (TRE) are primarily utilised to determine image similarity in registration and segmentation tasks. Overall, similarity metrics, such as Structural Similarity Index Measure (SSIM), indicate image quality by assuming the reference image is “perfect”, allowing for comparison of image quality.

**Table 1:** Descriptions of common full-reference image metrics that are used in literature, given two images (often a reference image and a “distorted” image). Full-reference refers to the measurement of the metric based on the reference (target or original) image.[95]

Metric	Description	Desired value
Peak Signal-to-Noise Ratio (PSNR)	Ratio of the maximum possible value (power) of a signal in one image, and the power of noise between the two images, measured in decibels (dB)	Higher
Contrast-to-Noise Ratio (CNR)	Ratio of the difference in signal between two areas of an image and the standard deviation of the image noise	Higher
Mean Absolute Error (MAE)	Represents the average of the absolute difference between corresponding pixels in two images	Lower
Mean Squared Error (MSE)	Measures the average squared difference between corresponding pixel values in two images	Lower
Root Mean Squared Error (RMSE)	Square root of MSE; measure the standard deviation of residuals	Lower
Structural Similarity Index Measure (SSIM)[90]	Measures image similarity via perceived change in structural information – luminance, contrast, and structure – between two images. Results in a decimal value between -1 and 1, where -1 is no correlation and 1 is perfect similarity	Higher
Multi-Scale Similarity Index Measure (MS-SSIM) [91]	Generalises the SSIM by using image information from several spatial resolutions. The luminance information at the highest resolution is combined with the contrast and structure information of multiple downscaled resolutions. Values closer to 0 indicate poor similarity, while values closer to 1 indicate better similarity	Higher
Feature Similarity Index Measure (FSIM)[96]	Measures the similarity between mapped features in two images. It utilises two variables: similarity in phase congruency feature maps and similarity in gradient magnitude feature maps. The former is derived from the detection of features in the image, while the latter is the computation of the image gradient	Higher
Noise Quality Measure (NQM) [97]	Measures similarity between two images with respect to the residual noise content. Based on modelling of additive noise perception in human visual systems	Higher
Visual Information Fidelity (VIF)[98]	Based on Natural Scene Statistics (NSS) and Human Visual System (HVS). Ratio between the information contained in the reference image and the information in the distorted image, after passing each through a channel in the HVS model	Higher
Dice Similarity Coefficient (DSC)	Measures similarity between two images. Results in a value between 0 and 1, where 0 indicates no similarity, and 1 indicate perfect similarity	Higher
Hausdorff Distance (HD)	Calculates the maximum distance (often in millimetres (mm)) between corresponding boundary values in two datasets. Used in medical image segmentation to measure the similarity between the original image and region of interest delineation (segmentation)	Lower
95% Hausdorff Distance (HD95)	Represents the 95th percentile of the calculated Hausdorff distance, used to minimise influence of outliers	Lower
Target Registration Error (TRE)	Determines the similarity of two images after a rigid or deformable registration by measuring the distance between two corresponding points	Lower

Studies in medical imaging often utilise metrics such as Mean Absolute Error (MAE) and Peak-Signal-to-Noise Ratio (PSNR) to quantify the image similarity and quality. However, there is some discussion around the acceptability of these measures, with some being observed to better represent human visual perception. Image “perceptual metrics”, such as Structural Similarity Index Measure (SSIM) [90], Multi-Scale Similarity Index Measure (MS-SSIM) [91], Feature Similarity Index Measure (FSIM) [92], High Dynamic Range-Visual Distance Predictor-2 (HDR-VDP-2) [93], and Learned Perceptual Image Patch Similarity (LPIPS) [94], have been developed to overcome the shortcomings of per-pixel measures.

#### Image enhancement: denoising, super resolution, and image generation

Image enhancement aims to improve image quality by boosting the information of interest in images, and possibly suppressing irrelevant information so that they are optimal for a particular application [99]. A variety of areas, including medical imaging, employ enhancement techniques to reduce noise, as well as increase contrast and resolution. Conventionally, enhancement techniques are based in the spatial and frequency domains. The spatial domain is the visual representation of the image, given by pixel intensity and location. Image enhancement in the spatial domain directly modifies these pixels through techniques such as histogram manipulation [100], Retinex algorithms [101], fuzzy set theory [102], and deep learning. Alternatively, the image may be manipulated in the frequency domain following a Fourier transform, governed by the rate of change in pixel intensity. High- or low-frequency components in the image can be filtered using a mask [103]. This is a natural state for CT data, as it is frequently analysed in the frequency domain through a sinogram, which presents the projections collected along the acquisition path. As such, some approaches utilise the CT sinogram as network input [104].

An important focus in image enhancement is the signal-to-noise ratio, which describes the balance between the meaningful data and meaningless noise. Enhancement can be achieved by increasing this ratio; minimising noise (denoising), while preserving original features such as edges and textures, improving contrast, and avoiding the generation of new artifacts [105]. Image noise impacts the interpretation of the image by distorting details or features with inaccurate pixel values [106]. Denoising is integral to the processing stage of image reconstruction, traditionally utilising filter algorithms or reconstruction techniques in either the spatial or frequency domains [107]. For example, block-matching and 3D filtering (BM3D) is a popular algorithmic denoising technique, but suffers from high complexity, inflexibility, and incomplete elimination of artifacts [108]. More recently, deep learning methods, such as neural networks (e.g. U-Net), have been utilised to overcome these issues [109].

Super resolution image enhancement methods are similar to denoising, but instead aim to increase the spatial resolution of images algorithmically, which is normally limited by the imaging system. Two types of super resolution method can be identified: single-image and multi-image. The objective of the former is to produce a high-resolution image from a single low-resolution image, whereas the latter uses multiple source low-resolution images [110]. Typical super resolution methods are computationally inefficient, complex, and require fine parameter-tuning, such as those discussed in Farsiu et al. [111]. Deep learning approaches have also been developed to address super-resolution problems [112].

Image generation or synthesis is the creation of new images from existing data and finds application in medical imaging predominantly in image-to-image translation and data augmentation tasks. Image-to-image translation aims to transform the contents of an image to a desired style, often used for modality translation (e.g. MRI to CT, PET to CT) [113]. Data augmentation involves generating realistic synthetic images for use in training neural networks, where datasets in medical imaging are often lacking in volume, diversity and quality [114]. Generative Adversarial Networks (GANs) are inherently suitable for this task. The generator network in a GAN is trained to synthesise a realistic desired output using an input, while the discriminator guides it [44]. The term “image generation” can refer to techniques attributed to other image enhancement tasks (denoising, super-resolution etc.), as well as image synthesis methods. However, in this review it is used to solely refer to image synthesis.

#### Image analysis: registration and segmentation

Both registration and segmentation methods are utilised across a variety of imaging fields but appear in the analysis stage of medical image processing [115]. These techniques are based on the image contents and how they can be processed for better examination.

Image registration focusses on geometrically aligning two or more images using corresponding intensity patterns (intensity-based methods) or image features (feature-based methods). One of the images is designated the “fixed” or target image, which remains the same throughout the process, while the other image is “moving”, as it is transformed to align with the target image [116]. The goal is to determine the coordinate transform such that the moving image and the fixed image are at the same position. There are two groups of geometric transformation used commonly in medical imaging: linear and non-linear. Rigid transformations align the two images through rotation, translation or reflection, while preserving spatial dimensions. Affine transformations introduce scaling and shearing, which alter the shape and size of the moving image. Non-linear or “deformable” transformations distort the moving image beyond linear registration techniques to achieve accurate alignment [117].

Image segmentation aims to divide an image into two or more distinct parts with reference to its contents. There are two categories of segmentation: semantic and instance. The former identifies and assigns a label to individual pixels within an image with respect to the pixel characteristics, forming ROIs that correspond to objects. The latter performs the same task as semantic, but instead delineates distinct instances of each object within the image [118]. Segmentation is integral to medical imaging and radiotherapy, as its used to delineate bone and tissue boundaries, creating planning target volumes (PTVs) etc., and is often performed manually by a clinician or automated algorithms. This process is generally labour intensive and time-consuming, making it an ideal task for developments in deep learning [119].

### Applications of deep learning in computed tomography (CT)

The applications of deep learning in CT outlined in this review include image denoising, super resolution, generation, registration, and segmentation. These applications are briefly explained prior to summarising some select recent publications, which stand as examples of approaches which have been developed in parallel to achieve similar goals. Tables 2 and 3 present an overview of the summarised publications for comparison, including the reported results for peak signal-to-noise ratio (PSNR), structural similarity index measure (SSIM), Dice similarity coefficient (DSC), and Hausdorff distance (HD) where applicable.

**Table 2:** Summary of deep learning approaches utilised in improving the pre-processing (enhancement) stages of reconstructed CT images described in this review. All values have been collected from their respective publications, representing the mean and standard deviation (where given) of the experimental results for the peak signal-to-noise ratio (PSNR) and structural similarity index measure (SSIM)

	Model Name	Author (Year)	Model Type	Dataset Type	PSNR (dB)	SSIM
Denoising	SGDNet	Huang (2022)	CNN	Abdominal	32.54 ± 1.16	0.9066 ± 0.0263
	CTformer	Wang (2023)	Transformer	Abdominal*	–	0.9120
	SIST	Yang (2023)	Transformer	Head, Chest, Abdominal	41.80 ± 1.37	0.9160 ± 0.0120
	CoreDiff	Gao (2024)	DDPM	Abdominal*	43.92 ± 1.33	0.9744 ± 0.0087
Super Resolution	GHASR	Chi (2022)	Transformer	Abdominal	39.28 ± 1.00	0.9781 ± 0.0060
	TextureWGAN	Ikuta (2023)	GAN	Chest	28.06 ± 0.44	0.9300 ± 0.0100
	PIQE	Nagayama (2023)	CNN	Cardiac	–	–
	LIT-Former	Chen (2024)	CNN + Transformer	Abdominal*	43.10 ± 1.25	0.9774 ± 0.7100
Image Generation	SCECT cGAN	Chun (2022)	CNN	Cardiac	21.57 ± 1.85	0.7700 ± 0.0600
	Comp-GAN	Zhao (2023)	CNN	Head and Neck	26.50 ± 1.00	0.9400 ± 0.0200
		Yang (2023)	Transformer	Head and Neck	31.20 ± 2.30	0.8500 ± 0.0400
	MC-IDDPM	Pan (2024)	Transformer + DDPM	Head	26.49 ± 2.81	0.9470 ± 0.0320

^*^ 2016 NIH-AAPM-Mayo Clinic LDCT Grand Challenge dataset [120]

**Table 3:** Summary of deep learning approaches utilised in improving the processing (analysis) stages of reconstructed CT images described in this review. All values reported as stated in the original publications, representing the mean and standard deviation (where given) of the experimental results for the Dice similarity coefficient (DSC) and Hausdorff distance (HD)

	Model Name	Authors, Year	Model Type	Dataset Type	DSC	HD (mm)
Registration	UDAN	Hu (2022)	CNN	Chest	0.8634	–
		Schaefferkoetter (2023)	CNN	Whole Body	–	–
	Patch-RegNet	Zhao (2023)	CNN + Transformer	Head and Neck	0.7600 ± 0.0500	–
	CMAN	Pham (2024)	CNN	Abdominal	0.9460 ± 0.0160	44.73 ± 20.80
Segmentation	nnU-Net	Li (2022)	CNN	Cardiac	0.9698 ± 0.0081	–
	3D U-Net + 2D PatchGAN	Colbert (2023)	CNN	Chest	0.9800 ± 0*	2.25 ± 1.09*†
	Absegnet	Liao (2023)	CNN	Abdominal	0.8565	10.18†
	GWO-SwinUNet	Kumar (2024)	CNN + Transformer	Abdominal	0.9870	2.00†

^*^ No average value reported in original publication, the best result (left lung) was selected for presentation

^†^ HD95 value reported

#### Image enhancement – denoising

In x-ray-based imaging, the number of incident photons on the sensor is directly related to the image noise; more photons result in less statistical noise. This relationship introduces a problem in medical imaging modalities such as CT, where increasing the number of photons results in higher ionising radiation dose to the patient but reduces the noise in the reconstructed image [121]. Low-dose CT (LDCT) technology has gained interest due to the reduced risk compared to normal-dose CT imaging, however, a lower dose is inherently afflicted with increased noise, affecting the diagnostic capability. The noise present in CT imaging is signal dependent and difficult to reduce using conventional image denoising techniques. Traditional non-AI based methods, such as sinogram-domain filtering, iterative reconstruction, and post-processing techniques require greater user-input and computational cost [109].

In their work on LDCT, Huang et al. [122] proposed a method of laterally combining two subnetworks: a structural semantic extraction (SSE) subnetwork, and a 3D denoising subnetwork, into a main framework, the segmentation-guided denoising network (SGDNet). The SSE subnetwork consists of a 3D convolutional encoder-decoder architecture and is tasked with predicting three structural semantic regions (subcutaneous fat, muscle, and visceral fat). The goal of the denoising subnetwork is to estimate the normal-dose CT images using the structural semantic information provided by the SSE subnetwork. A novel structural semantic loss was also introduced for network training. Segmentation labels for subcutaneous fat, muscle, and visceral fat were provided for simulated LDCT clinical abdominal images sourced from thirty-five healthy patients. The performance of the SGDNet was compared to three common networks: a convolutional neural network (CNN) [123], residual encoder-decoder CNN (RED-CNN) [124], and self-attention CNN (SACNN) [125], and evaluated using peak signal-to-noise ratio (PSNR), structural similarity index measure (SSIM), and normalised root-mean-square error (NRMSE), and the Dice similarity coefficient (DSC) for each structural semantic region. Quantitative results indicated that the proposed SGDNet achieved the best performance among the compared networks for all image metrics, as well as the highest DSC for all structural semantic regions. Although the proposed method shows improvement over other networks, the authors acknowledge the impact of abnormalities within CT images, as well as incorrect segmentation, on the network outcome.

To overcome the inability to adequately capture global context information, Wang et al. [126] addressed the issue of increased noise in LDCT by incorporating an alternative architecture to the well-known CNN, a convolution-free Token2Token dilated vision transformer (CTformer). The proposed CTformer model follows a central residual encoder-decoder structure comprised of a tokenisation-detokenisation, a transformer, and Token2Token dilation blocks. The tokenisation block “unfolds” the 3D CT volume into a sequence of two-dimensional patches (or tokens) before the transformer blocks carry out the attention process, which allows the network to focus on the importance of features it identifies. Finally, the Token2Token dilation blocks utilise cascade tokenisation to model local information and feature representation. A dataset consisting of ten patients receiving abdominal CT imaging was used for training and testing, including three slice thicknesses at both normal- and low- dose levels. The parameters of the CTformer were quantitively compared against four models – RED-CNN, Wasserstein GAN-Visual Geometry Group (WGAN-VGG) [127], modularised adaptive processing neural network (MAP-NN) [128], and attention-guided denoising neural network (AD-NET) [129] – for SSIM and root-mean-square error (RMSE). Wang et al. determined that their proposed method produced the best results for both SSIM and RMSE among the networks tested, despite the CTformer possessing fewer trainable parameters. This ultimately results in a model that is efficient at denoising, outperforming other methods while having lower computational cost. However, the resultant denoised images were observed to possess an “oversmoothness”, which may inhibit diagnostic viability. To improve the textural quality, the authors have suggested incorporating both perceptual and adversarial losses.

Another study that evaluates the effectiveness of incorporating a vision transformer was conducted by Yang et al. [130], however, their proposed model focusses on denoising in the sinogram domain instead of the image domain. Traditionally, a vision transformer creates the model input by separating a two-dimensional image into individual tokens, however, a sinogram is a combination of several view angles of projection data and would suffer data inconsistency if treated likewise. Therefore, the study proposes the sinogram inner-structure transformer (SIST) to extract feature information from the sinogram domain on a per-angle basis for CT images of the head, chest, and abdomen. Within the sinogram domain there are corresponding point pairs which possess the same values, leading to a predictable conjugate structure (called the inner structure) that may be interrupted by random noise. The study also introduces a custom loss function to account for local and global inner-structure, as well as an image reconstruction model to complete further denoising once the data is transformed into the image domain. The SIST was qualitatively assessed for PSNR, SSIM, and RMSE against a variety of denoising methods, including: filtered backprojection (FBP), iterative reconstruction (penalised weighted least squares edge-preserving regulariser (PWLS-EP), penalised least-squares union of learned transforms (PWLS-ULTRA)), and CNN-based (RED-CNN, domain-progressive residual network (DP-ResNet) [131]). Experimental results revealed that the SIST model had the best performance among the methods compared. The DP-ResNet CNN model, despite using a similar dual-domain (sinogram- and image-based) approach, possessed lower results than the proposed model and completed processing in × 4.4 the time, indicating the positive impact of transformer architecture. This mirrors the findings in Wang et al. [126], which indicates the success of vision transformers in efficiently denoising LDCT images.

A different approach to denoising LDCT imaging was investigated by Gao et al. [132], who introduced a novel generalised diffusion model, CoreDiff, combined with a secondary CNN-based restoration network, CLEAR-Net. Traditional generalised or “cold” diffusion methods are latent variable generative models which apply a variety of degradation transformations (e.g. blurring, noise, pixel masking, downsampling etc.) to the clean input image (cold state) to create a completely degraded image (hot state). Then, the reverse process is undertaken, allowing the model to learn a probability distribution from which synthetic images may be sampled from. Gao et al. modify the process by utilising LDCT as the endpoint instead of a sample of pure Gaussian noise, requiring a smaller number of sampling steps compared to the traditional model. A generalised degradation operator was also introduced, with the form D(xo…), to address CT number deviation in intermediate noisy samples and align more closely to the observed degradation of CT images due to dose reduction. The CoreDiff model was compared against four methodologies for LDCT denoising: iterative reconstruction (PWLS), CNN-based (RED-CNN, parameter-dependent framework-RED-CNN (PDF-RED-CNN) [133]), GAN-based (WGAN-VGG, dual-domain U-Net-based GAN (DU-GAN) [134], content-noise complementary learning-U-Net (CNCL-U-Net) [135]), and diffusion-based (denoising diffusion models for denoising diffusion MRI (DDM^2^) [136], improved denoising diffusion probabilistic models (IDDPM) [137]). Qualitative evaluation of quarter-dose abdominal CT data was conducted by calculating the PSNR, SSIM, RMSE, feature similarity index (FSIM), visual information fidelity (VIF), and noise quality metric (NQM). It was found that the proposed model outperformed all compared methods, however, the authors note that the degradation transformation operator requires further refinement to account for other noise contributions such as patient motion, metallic artefacts etc.. Similarly, CoreDiff also suffers from a long inference time compared to the CNN- and GAN-based methods.

#### Image enhancement – super resolution

While often higher than other medical imaging modalities, spatial resolution in CT can be affected by motion, scan time and radiation dose, notably in low-dose CT [138]. In low-dose CT a comparatively smoother kernel is used to minimise the effect of noise, however, this often reduces the spatial resolution. Due to better reconstruction of high-frequency information like texture and edges, deep learning techniques can succeed where traditional mathematical models suffer in improving resolution. Sharing a similar goal to denoising, deep learning super resolution methodology was first proposed in 2014 by Dong et al. [139], which utilised a three-layer convolutional neural network (CNN) to map between low- and high-resolution (LR and HR, respectively) images.

In a study by Chi et al. [140], the impact of combining a hybrid attention mechanism with global feature fusion on abdominal CT image super resolution was investigated. As super resolution tasks focus on preserving edges and textures, the proposed global hybrid attention super resolution (GHASR) network takes advantage of attention mechanisms to better resolve different levels of feature frequency. Feature extraction is guided by a module consisting of a stack of Swin Transformer blocks, which feed into a multi-branch hierarchical self-attention module (MHSM), and a multidimensional local topological feature enhancement module (MLTFEM). The MHSM performs several operations to the hierarchical features extracted by the core transformer blocks, mapping multiple feature levels and establishing a connection between these features using a self-attention mechanism. Deep features are also fed into the MLTFEM, which aims to filter out features specific to undesired regions, therefore improving the efficiency of extracting meaningful information corresponding to ROIs. They calculated the metrics PSNR and SSIM at three scale factors, quantitatively comparing the proposed GHASR network against nine methods: bicubic interpolation, enhanced deep super resolution network (EDSR) [141], residual channel attention network (RCAN) [142], enhanced super resolution GAN (ESRGAN) [143], holistic attention network (HAN) [144], internal graph neural network (IGNN) [145], soft-edge assisted network (SeaNet) [146], non-local sparse attention (NSLA) [147], and SwinIR [148]. GHASR was evaluated using the abdominal 3D-IRCADb dataset at a scale factor of two, resulting in 16.1 million trainable parameters, compared to 65 million in the original study proposing transformer models by Vaswani et al. [50]. The second-best PSNR results for the same dataset and scale factor were obtained using the RCAN model, which may have been limited by a small receptive field and inferior global information attention capabilities. The proposed model successfully produces super resolution CT images; however, it also required a relatively long training time of 37 h.

To combat over-smooth output images often generated by convolutional neural networks (CNN), a study conducted by Ikuta and Zhang [149] introduces a multitask regulariser (MTR) to a Wasserstein generative adversarial network (WGAN), called TextureWGAN. The primary cause arises from the mean-squared error (MSE) loss incorporated into model training, which fails to adequately capture spatial information like image texture. Preserving realistic image texture and pixel fidelity (accuracy) is important in diagnostic quality, especially in low-contrast regions where over-smoothing can reduce fine structures. The performance of the generator network within a GAN model is highly dependent on the loss functions and regularisation parameters. In the TextureWGAN generator, the MTR utilises an adaptive regularisation scheme with both MSE and perception loss, as well as an image pair (true and generated) to avoid loss of pixel information. Traditional WGAN generators take a latent vector as input, whereas the TextureWGAN “guides” the training process by taking both true and generated images as input, ensuring that there is a high correlation between the ground truth and synthetic output. After training on a dataset of chest CT images, the TextureWGAN model was compared to FBP, non-local mean filter (NLM) [150], total variation (TV) regularisation [151], U-Net (CNN), and the standard WGAN, using PSNR and SSIM, as well as first- and second-order statistical texture analyses. Although the TextureWGAN and U-Net models produced output images with similar PSNR and SSIM, the latter, CNN-based model generated results without appropriate texture. The standard WGAN model, comparatively, produced images with good texture preservation, but failed to generate images with structural correlation. The proposed TextureWGAN shows that preserving the original image texture does not have to come at the cost of accuracy in pixel values while using MSE loss.

Alongside academic developments in improving the image quality of reconstructed CT images, commercially available or vendor-specific deep learning models have also appeared. However, these models still require clinical evaluation prior to implementation. Nagayama et al. [152] investigated the effect of a commercially available deep learning reconstruction algorithm, the Canon Medical Systems Precise IQ Engine (PIQE), to achieve super resolution on low-dose coronary CT angiography (CCTA) for coronary artery disease (CAD). Due to the dimensions of the cardiac structures imaged, CCTA requires precise spatial resolution for accurate angiogram interpretation. Imaging with an ultra-high resolution CT scanner can improve the spatial resolution, however, the reconstructed images suffer from increased image noise and temporal artifacts, leading to lowered diagnostic quality. To evaluate the performance of the PIQE model, Nagayama et al. calculated the image noise and contrast-to-noise ratio (CNR) for processed images of four major cardiac structures. Quantitative comparison of the PIQE algorithm (SR-DLR) was completed against hybrid iterative reconstruction (HIR), model-based iterative Reconstruction (MBIR), and normal-resolution deep learning reconstruction (NR-DLR). Overall, the SR-DLR had the lowest image noise among the reconstruction methods, as well as the highest contrast-to-noise ratio. Blooming artefacts due to plaque calcification were significantly lower using the SR-DLR method than in NR-DLR, HIR, and MBIR. Qualitatively, the SR-DLR also achieved the best scores from the two radiologists in cardiac structure delineation, noise magnitude, noise texture, image sharpness, edge smoothness, and overall quality. Despite performance, PIQE and other commercial models are examples of “closed”, meaning that model parameters cannot be adjusted as easily.

The noise of low-dose CT (LDCT) can be reduced by using a larger slice thickness and slice interval, however, this decreases the resolution longitudinally. Chen et al. [153] proposed the link in-plane and through-plane transformer (LIT-Former) to perform simultaneous denoising in-plane and deblurring through-plane for abdominal 3D CT images. LIT-Former extracts both local and global information by combining vision transformer and convolutional networks in a U-shaped framework, incorporating multi-head self-attention module (eMSM) and convolutional feed-forward network (eCFN) blocks. For the eMSM, the in-plane denoising is completed using a transposed attention operation and multi-head attention in the channel dimension prior to the key-query dot product, whereas the through-plane deblurring is completed using a standard attention operation and standard multi-head attention. To capture contextual information, the eCFM performs 3D convolutions by way of a 2D in-plane convolution (for the denoising task) followed by a 1D through-plane convolution (for the deblurring task). A dataset comprising of low-dose (quarter) abdominal CT images was utilised, where normal-dose CT images of 1mm slice thickness were used as the ground truth and LDCT images of 3mm slice thickness were used as model input. Quantitative performance of the proposed model was evaluated through calculating the PSNR, RMSE, SSIM (3D), and SSIM (2D), comparing with alternative super resolution methods: 3DU-Net, RED-CNN, edge enhancement-based densely connected CNN (EDCNN) [154], improved DenseNet and deconvolution-based network (IDD-net) [155], temporal adaptive module TAM [156], temporally-adaptive convolutions (TAdaConv) [157], and BasicVSR +  + [158]. Across all image metrics the LIT-Former model achieved the best results, mirroring the success of implementing a transformer network for super resolution in Chi et al. [140]. A version of the LIT-Former without the eMSM block, called (2 + 1)DUnet, was also tested to determine the impact of including module. For PSNR and both SSIM results, the (2 + 1)DUnet performed second-best, indicating the viability of the LIT-Former architecture, with or without the eMSM block.2.2.3 Image Generation – Synthetic CT

####  Image generation – synthetic CT

Increasingly, radiotherapy involves multi-modal imaging data such as MRI, PET, or CT. For example, MRI is becoming a central part of radiotherapy imaging due to three main factors: the lack of ionising radiation and associated dose, superior soft-tissue contrast, and functional imaging capabilities. However, MRI does not possess the relative electron density data that is present in CT [159]. This relates the visual representation of anatomy in the reconstructed images to the density of the corresponding tissue, allowing for accurate identification. Due to this, radiotherapy treatment planning utilising only MRI is less developed than with clinical CT imaging. Synthetic image generation via a deep learning network allows the data from the source domain (e.g. MRI) to be transferred to the target domain (e.g. CT).

In MRI-based radiotherapy planning, the field of view (FOV) is often reduced to minimise imaging time and compensate for peripheral region geometric distortion, resulting in images that are truncated in those areas. Due to missing anatomical features, significant dose calculation errors may arise in MRI-only treatment planning that uses synthetic CT images generated using truncated MRI. In a study by Zhao et al. [160], a compensation cycle consistent GAN (Comp-GAN) was trained to generate synthetic CT images that successfully predict missing anatomy, namely the occipital region of the skull. The architecture of the Comp-GAN model is a modified CycleGAN [161] model, consisting of two residual-U-Net generators and two convolution-based discriminators. Three types of clinical head-and-neck images are utilised for model training: the original non-truncated MRI, manually truncated MRI, and paired CT. Generated images produced by the Comp-GAN model were quantitatively compared against the original CycleGAN model using MAE, PSNR, and SSIM. A statistically significant improvement in all three metrics was observed using the Comp-GAN model. The authors also extended the study by comparing the effect of incorporating body contour information into the model input, which were created using the clinical CT images and rigidly registered to the corresponding MRI. Results for MAE, PSNR, and SSIM were similar to the original Comp-GAN model, however, the inclusion of body contours improved the shape and structure of the truncated region in resultant synthetic CT images. When incorporating body contour information into the model input, the mean square distance (MSD) decreased and the DSC increased, indicating the improvement in the accuracy of synthesising missing soft-tissue and bone. However, the authors identify a drop-off in anatomical accuracy in generated images as truncated regions increase in size, as critical structures may remain missing or distorted. Contrast-enhanced CT (CECT) may be used to better visualise cardiac substructures for radiation-induced risk assessment, however, guidelines for acquisition of planning CT for breast cancer patients does not involve administration of a contrast agent. Chun et al. [162] developed a deep learning methodology which uses a conditional generative adversarial network (cGAN) that aims to generate synthetic contrast-enhanced CT (SCECT) using non-contrast CT (NCT) data. Their cGAN architecture consisted of a two-dimensional fully convolutional DenseNet (FC-DenseNet[163]) generator network, alongside a modified PatchGAN discriminator network. Unlike a standard DenseNet, FC-DenseNet only upsamples feature maps created by the preceding dense block, allowing the network to restore the resolution of the initial input. The authors evaluated the image quality of the output SCECT images using mean absolute error (MAE), PSNR, and SSIM, as well as clinical viability assessment through contouring and dosimetry. For the generated SCECT images, the calculated MAE, PSNR, and SSIM were found to be statistically significantly different from the input NCT images. Contouring and dosimetry results of the SCECT images were successful and not significantly different from the ground truth CECT images. However, due to the inconsistent nature of contrast projection procedure, use of CECT as a ground truth produced variations in Hounsfield Unit intensity in resultant SCECT images. To resolve this, the authors trained the model to develop a contrast averaging feature, which balances the inaccurate brightness of structures observed.

Cone-beam CT (CBCT) is often utilised in image-guided radiotherapy (IGRT), through an onboard unit mounted on a Linac gantry, as it acquires a volumetric image of the patient in the treatment position, ideal for intra-fractional corrections. However, the inherent characteristics (e.g., beam geometry, slow gantry rotation, scatter etc.) of CBCT imaging leads to increased artefacts, reducing the overall image quality and increasing variation in HU [79]. As convolution-based models learn in pixel-wise manner and CBCT image noise is not pixel-specific, Yang et al. [164] proposed a Swin transformer-based model that incorporates an encoder-decoder structure with a hybrid loss function. Similar to the MRI-to-CT image translation task, CBCT data (source domain) is utilised to create synthetic CT images (target domain) which may be used in adaptive radiotherapy treatment. The use of a transformer-based model avoids the noise propagation in pixel-based convolutions, as it learns to identify long-distance dependencies (intra-layer and intra-tissue). Self-attention also reduces the persistence of noise and artefacts by training to suppress irrelevant information while emphasising relevant anatomical data. The proposed hybrid loss function incorporates three terms: masked MAE, SSIM, and gradient loss. Mask-weighted MAE loss is utilised to reduce variation in CT number, as well as reduce the MAE in bone tissue, as the ground truth CT and CBCT are well aligned in those regions. The proposed model was evaluated quantitatively against three convolution-based models (modified versions of CycleGAN [161], Pix2Pix [45], and a multi-resolution deep ResNet) for MAE, PSNR, and SSIM. Resultant synthetic CT images generated from noisy CBCT data were observed to have Hounsfield Unit values closely resembling corresponding clinical CT, suggesting viability in future clinical implementation. An additional “personalised” workflow was also introduced, allowing the model to fine-tune training with a single patient dataset after generalisation, improving the quality of output images for that specific patient.

GAN-based models are known to suffer from unstable training, inhomogeneity in output synthetic images, and extensive time to balance generator and discriminator hyperparameters due to their adversarial nature. To avoid these problems, Pan et al.[165] developed a transformer-based improved denoising diffusion probabilistic model (MC-IDDPM) to learn 3D image generation to create synthetic CT images from brain and prostate MRI data for radiation treatment planning. Different to the process described in Gao et al. [132], the MC-IDDPM follows a standard method consisting of a forward diffusion that adds only Gaussian noise and reverse diffusion that removes it. However, the proposed model introduces a Swin-Vnet for reverse diffusion and reduces the number of reverse diffusion timesteps by predicting both the mean and the variance. An additional MRI scan is utilised to condition the reverse diffusion process, guiding the creation of synthetic CT such that they do not deviate from the expected patient anatomy. To assess the capability of the proposed transformer-based diffusion model, Pan et al. compared it to both GAN-based (MRI-to-CT pixel-to-pixel GAN (MR-GAN) [166] and MRI-to-CT cycle-consistent GAN (MR-CGAN) [166]) and diffusion-based (2D-IDDPM [137], 3D-DDIM [167], and 3D-DDPM [168]) models, calculating the MAE, PSNR, multi-scale SSIM, and normalise cross-correlation (NCC). Among the compared methods trained on a brain dataset, MC-IDDPM produced the most authentic synthetic CT images with well-preserved anatomical structures and reduced noise. Subsequent dosimetric analysis of treatment plans generated using the synthetic CT images showed dose differences within ± 0.34% across four planning target volumes (PTVs), indicating close alignment with corresponding planning CT. However, the authors acknowledge a significantly high processing time (approx. 12 min) compared to GAN-based methods (approx. 20 s), caused by the large number of iterations in generating images inherent to the diffusion method.

#### Image registration

A range of deep learning methods have been investigated to assist with the alignment of medical images, focussed on estimating similarity/dissimilarity metrics or predicting transformation parameters. Similarity metrics describe how similar two images are, while transformation parameters describe the types and magnitude of the transformation required to align the two image coordinate systems.[169] Recent deformable image registration methods predominantly focus on using neural networks to improve the entire registration process, rather than the similarity metric alone [116].

While both GAN and U-Net models have been successful in medical CT image registration, CNN-based networks often inadequately represent long-range spatial relationships (connections between non-adjacent objects), especially in multi-modal registration. Building from previous studies, Hu et al. [170] proposed an unsupervised dual attention network (UDAN) for chest CBCT-CT image registration, consisting of a U-Net-like network to extract features, and a dual attention module (DAM). The DAM is introduced into the bottleneck of the network to utilise high-dimensional features with different scales and is comprised of a scale-aware position attention block (SP-BLOCK) and a scale-aware channel attention block (SC-BLOCK). The former selectively integrates the weighted sum of the features from all positions into the multi-scale features, while the latter consolidates the features from different channels with respect to the structural relationship between channels. Rui et al. compared their proposed model against six existing methods – three using standard deformable registration (advanced normalisation tools (ANTs) [171], Elastix [69], and B-Spline non-rigid registration [172]), and three deep learning based (VoxelMorph) [173], volume tweening network (VTN) [174], and CycleMorph [175]) – evaluating the SSIM, DSC, and target registration error (TRE). A statistically significant increase in DSC and SSIM, and decrease in TRE was observed, showing the improvement in registration due to the attention blocks. A variant of the UDAN model incorporating a simplified DAM also revealed the effectiveness of utilising dilated convolution operations with residual structure in the SP- and SC-BLOCKs, reducing inaccurate feature matching resulting from voxel intensity differences between CBCT and CT images.

In PET-CT imaging, CT scans are utilised to generate an attenuation correction (AC) µ-map, however, both physiological motion and patient movement between consecutive scans negatively impacts the spatial alignment of the AC map to PET, leading to artefacts in reconstructed PET images. Reducing these motion-induced artefacts in whole-body PET/CT imaging is the focus of a study by Schaefferkoetter et al. [176], which introduces a CNN-based method comprising a U-Net-like feature extractor followed by a displacement vector field (DVF) regressor. Using a supervised approach, the feature extractor creates feature maps by taking as input an image pair consisting of CT warped by a randomly generated motion field to simulate movement distortion, and the corresponding PET. These output feature maps are then input into the DVF regressor network, which aims to predict a 3D DVF which characterises the relative deformation of the original PET-CT image pair. The generated DVFs are then used to elastically warp the anatomical image (CT) to spatially align with the corresponding functional imaging (PET). Performance of the networks was evaluated by comparing the image-pairs which have been corrected by the network-generated AC µ-map to image-pairs which have been registered using an uncorrected AC µ-map. This was completed through quantitative assessment of the MSE of the DVFs, the MAE between the AC µ-maps, and the MAE of the AC PET reconstruction. The corrected, re-aligned images showed an improvement in all tested metrics, reducing the DVF MSE by (52.78 ± 21.90)%, the µ-map MAE by (50.00 ± 9.32)%, and the PET MAE by (88.07 ± 34.30)% for a single patient subject. The authors note that, despite the success in correcting misalignment between the AC µ-map, and the PET imaging quantitative analysis is limited by the absence of a non-simulated ground truth, as one input for the feature extractor is a CT image distorted by simulated motion.

Multimodal deformable registration between MRI and CT imaging faces some challenges with head and neck radiotherapy misalignment in extra-cranial sites, caused by contrast differences and anatomical changes between scans. Zhao et al. [177] developed a framework (Patch-RegNet) to perform CT-MRI deformable image registration (DIR) for head and neck MR-Linac treatments, utilising the proposed patch-based ViT-Morph model. The proposed network combines the CNN-based VoxelMorph [178] framework with a vision transformer to avoid the loss of long-range spatial information associated with CNNs. The Patch-RegNet workflow contains three registration stages: an initial whole-volume rigid registration, a patch-based local registration following the division of input images into overlapping patches, and a patch-based deformable registration via ViT-Morph. Applying these two registration processes before input into ViT-Morph ensures a favourable local pre-alignment between the images before DIR is attempted. Registration accuracy of the Patch-RegNet framework was quantitatively evaluated by determining the DSC and MSD across multiple organs in head and neck patients. These values were compared against three alternative methods: standard rigid registration, Monaco-based DIR, and VoxelMorph. With an improved DSC and MSD, Zhao et al. determined that their proposed Patch-RegNet framework and ViT-Morph network achieves an accurate and statistically significant improvement in inter-modality alignment of CT-MRI images. Further study of the ViT-Morph network without the Patch-RegNet framework revealed slight improvement in DSC and MSD compared to the VoxelMorph method but remained lower than with the framework included. As the ViT-Morph network is the final registration stage of three, this indicates that providing the network with well-aligned images produced via the prior global and patch-based local registration stages increases similarity and reduces misalignment.

Procedures including pre- and post-process CT imaging of the liver often require the use of traditional methods to perform deformable image registration due to the large deformations which commonly occur in the abdominal region. As these methods are time-consuming and inefficient, Pham et al. [179] developed an unsupervised cascaded multi-scale spatial channel attention-guided network (CMAN) for deformable registration of 3D liver CT images. A double coarse-to-fine strategy is utilised, consisting of a local coarse-to-fine registration and a global coarse-to-fine registration. Local coarse-to-fine registration is achieved through the base network, which follows standard U-Net design and outputs a deformation field that is the sequential warping of the local deformation fields in different resolutions. For each layer of the decoder stage, the feature map is refined by a channel-spatial attention block, consisting of two consecutive attention modules (channel and spatial). Stacking multiple base networks together (cascading) allows for global coarse-to-fine registration by further sequential warping, incorporating multi-layer contextual information into the final deformation field. Registration accuracy was assessed by comparing the DSC, average symmetric surface distance (ASSD), Hausdorff distance (HD), and TRE, against two traditional DIR algorithms (ANTs [171], elastix [69]) and three unsupervised networks (VoxelMorph [178], dual-stream pyramid registration network (DPRN) [180], VTN [174]). Although it was found that a five-fold cascaded CMAN model performed the best overall for intra-modal liver registration, a single CMAN network (uncascaded) produced better results than the alternative techniques. The effect of cascading mechanism was also evaluated, by varying the number of stacked CMAN networks the authors determined that the most significant improvement in registration accuracy occurs from one to two cascades. The success of the CMAN model indicates the effectiveness of incorporating cascading and attention mechanism into the workflow of abdominal CT image registration necessitating large deformation fields.

#### Image segmentation

In medical imaging, segmentation is used to localise regions of interest, such as specific anatomy or pathology such as tumours. Conventionally, segmentation is completed by using background image data and creating a mask, either through manual painting, or semi-automated thresholding algorithms. However, this process is often time-consuming. Like other image problems in computer vision, image segmentation benefits from convolutional neural networks, U-Nets, and adversarial networks.[118] Well-known segmentation tasks include brain and brain-tumour, lung, pulmonary nodules, liver and liver-tumour, as well as cardiac [181]. Several techniques and approaches to deep learning assisted image segmentation are discussed more broadly in Ghosh et al. [182].

Despite the success of deep learning approaches in medical imaging, segmentation of cardiac computed tomography angiography (CTA) remains challenging due to small, complex structures and image data heterogeneity. As preoperative simulation of transcatheter aortic valve intervention (TAVI) relies on accurate segmentation of the aortic valve and surrounding tissues, Li et al. [183] assessed the effectiveness of a 3D no-new U-Net [184] (nnU-Net) in cardiovascular CT image segmentation. The nnU-Net model is designed to perform supervised semantic segmentation without specialist knowledge or manual intervention, as it automatically configures itself depending on the input dataset. Configuration is based on three types of parameters: fixed, rule-based, and empirical. Fixed parameters are not adjusted with respect to the input dataset, whereas rule-based parameters use the characteristics of the training dataset to adapt properties of the model following hard-coded rules. Remaining parameters, such as U-Net configuration (2D, 3D etc.) and post-processing optimisation, are determined empirically. Ground truth labels for the dataset are created using the ITK-SNAP software, which semi-automatically segmented the aorta, Valsalva sinuses, coronary arteries, and left ventricle. Primarily, performance of the nnU-Net was evaluated through calculation of the DSC. DSC results for three recent studies were presented to compare the performance of the nnU-Net with alternative deep learning approaches, including a standard 2D U-Net, a 2D multi-view multi-network CNN, and a 3D U-Net. Although the parameters of these alternative methods have been tuned specifically for cardiovascular segmentation tasks, the nnU-Net produced a higher similarity to ground truth segmentation. Li et al. demonstrate the capability of the highly generalisable nnU-Net model in segmentation of the aortic valve and surrounding tissue without the manual parameter adjustment required of other models.

In breast cancer CT imaging, proximity to organs-at-risk (OARs), like the heart, lungs, lymph nodes, and contralateral breast, contribute to the need for high accuracy in segmentation and delineation, as this increases the risk of metastasis [185]. To address this issue, Colbert et al. [186] utilised two networks, a primary network consisting of a 3D U-Net, and a secondary network consisting of a 2D PatchGAN[45], for auto-segmentation of regions of interest (ROIs). Both the training and testing datasets included five manually segmented ROIs, left breast, right breast, left lung, right lung, and the heart, following clinical procedure. A pretrained residual network, ResNet(2 + 1)D-18, was used as the encoder for the 3D U-Net design for feature extraction, with a mirrored design for the decoder stage. Initial segmentation mask predictions made by the 3D U-Net were then corrected by the 2D PatchGAN network, aiming to compensate for the information loss that arises from CT scan downsampling, as well as the upsampling interpolation. After auto-segmentation, the Dice similarity coefficient (DSC), mean surface distance (MSD), and 95th-percentile Hausdorff distance (HD95) between the predicted and reference masks were calculated. These metrics were calculated for each of the ROIs and subsequently compared to previous studies in thoracic imaging of the same ROIs where possible. DSC, MSD, and HD95 values were all improved after mask correction by the 2D PatchGAN network. DSC results were better than previous studies for the left breast, left lung, and right lung, and achieved comparable results for MSD and HD95. The proposed pipeline, which includes a correction network following segmentation, achieved favourable results while maintaining a simple combination of 3D U-Net and 2D PatchGAN.

Abdominal CT imaging includes several OARs requiring accurate and individual delineation for dose evaluation in radiotherapy planning, however, many deep learning models are trained using homogeneous datasets. These datasets may only include images from a single scanner or a single centre, lacking generalisation. Liao et al. [187] proposed a deep learning model, AbsegNet, to produce accurate delineation of sixteen abdominal OARs trained using a large quantity of diverse data. A clinical dataset consisting of 300 labelled abdominal patient scans exhibiting a variety of tumour locations is used for training to avoid overfitting caused by highly-specific and limited data. AbsegNet leverages knowledge distillation, or “teacher-student” training, which involves the transfer of knowledge from a larger, more accurate network (the teacher) to a smaller, more efficient network (the student). The size of a model is dependent on the number of parameters, which defines the computational power required for operation, where more parameters necessitate more memory. During training, input images are augmented weakly via random noise for the teacher network and strongly via a random spatial or intensity transform for the student, before the output from the teacher network is used as input for the student network. The performance of the proposed model was compared against five alternative deep learning methods: 2D U-Net, 3D U-Net, DeepLabV3 + [188], Attention-UNet [189], and SwinUNETR [190], evaluated quantitatively for each OAR using volumetric DSC and HD95. When introduced with unseen heterogeneous datasets, such as those sourced from different hospitals, AbsegNet demonstrated a mean DSC of > 0.9 for eight OARs and < 0.8 for two OARs (duodenum and adrenals), and a mean HD95 of < 10mm for nine OARs and > 15mm for three OARs (colon, duodenum, and femur). The authors note that despite the successful performance of the proposed network the inaccuracy of the duodenum segmentation may arise from the complex anatomical structure of the organ itself.

In any medical image segmentation task, CNN-based deep learning networks can suffer from overfitting, noise sensitivity, and convergence, leading to low-quality results, especially in anatomically complex organs such as the liver. Kumar et al. [191] developed a hybrid model consisting of a grey wolf optimised Swin transformer with U-Net model for liver segmentation in abdominal CT imaging, called GWO-SwinUNet. Combination of Swin Transformer architecture into the U-Net design allows the model to better extract long-range dependencies and fine-grained spatial details due to self-attention mechanisms. The grey wolf optimiser (GWO) algorithm is a population-based meta-heuristics optimisation strategy based on the social hierarchy and hunting behaviour of grey wolves, where four types of solutions are presented: alpha (strong fit), beta, delta, and omega (weak fit). Optimisation is performed through mathematical modelling of three stages: searching, encircling, and attacking the prey (optimum), leading to improved parameter tuning compared to conventional optimisation strategies like Adam [192]. Following U-Net design, the encoder stage is comprised of alternating GWO-Swin transformer and patch merging layers to extract features. The decoder stage concatenates feature maps from corresponding layers of the encoder stage to preserve spatial information lost in the downsampling process. DSC and HD were calculated to quantitatively evaluate performance, comparing against a variety of U-Net-based models, including a standard U-Net and SwinUNet. For an unseen abdominal CT image dataset, the proposed GWO-SwinUNet model produced an increased DSC and a decreased HD, improving upon results produced by other methods and showing good network generalisation. GWO-SwinUNet demonstrated superior segmentation accuracy, as well as improvements in convergence speed and validation loss compared to the standard SwinUNet due to the inclusion of the GWO optimisation strategy.

## Challenges in applying deep learning

Deep learning has opened a variety of opportunities to improve medical imaging, including CT image enhancement, registration, and segmentation, however, some challenges limit its potential for future clinical implementation. The following sub-sections, model choice, computational demands, data availability, and clinical implementation, are presented as examples of issues broadly faced by developers and researchers in applying deep learning. Although more obstacles exist in its implementation, these have been selected as they indicate common areas where research gaps arise and must be addressed by further study.

### Model choice

The significant increase in deep learning models has resulted in a wide variety of choices for computer vision tasks, including those specific to medical image processing. Beyond an exploration of convolutional neural network (CNN) architecture, with U-Net and GAN as examples, this review briefly outlined several models utilised in recent literature. There is no hierarchy of overall model performance presented in this work, as model selection is intrinsically related to the task, dataset, and computational resources available.

CNNs are intrinsically suited to image processing tasks due to the convolution operation, which provides efficient and well-generalised feature extraction, however, it possesses a limited receptive field. The receptive field is the region of the input image each neuron within a layer observes, determined by the size and stride of the convolution kernel. CNN models are hierarchical in nature, as initial layers extract local, low-level features and later layers extract global, high-level features. This is reflected in the size of the receptive field, where initial layers have a small receptive field which increases in size with each layer. Any pixel information outside of the receptive field is not passed onto subsequent layers, leading to a limited capability to capture relationships between distant or non-adjacent data elements (long-range dependencies). CNNs fail to adequately connect information from widely separated input regions within an image, resulting in inaccuracy in model predictions [193].

Transformers (or vision transformers) can be utilised to address the limitations of CNN models, as they possess large receptive fields, allowing for a better understanding of more distant contextual information. They were originally introduced in 2017 for machine language translation by Vaswani et al. [50], but has been applied to a range of natural language and computer vision tasks. The fundamental component of the transformer is the self-attention mechanism, which aims to emphasise important features and supress unnecessary ones.

### Computational demands

The progress of deep learning is heavily reliant on computational power. Until the move to Graphical Processing Unit (GPU) processing in the 2010s, developers were limited to using fewer training samples and smaller-scale models [194]. As the number of layers in a deep neural network increases and features are extracted with greater complexity and abstraction, the number of network parameters also grows dramatically, leading to the need for higher computational requirements and more training data [195]. For example, the well-known AlexNet model proposed in 2012 consists of 60 million parameters and required two 3GB Nvidia GTX580 GPUs to train over five to six days [196]. Although GPUs have seen improvement, Thompson et al. [197] catalogued over one thousand research papers to determine the demand for computational power, concluding that it is rapidly becoming technically and economically unsustainable. This challenge may be addressed by the way of two routes: increase computational power or increase model efficiency. The former was addressed in Thompson et al., while the latter is a goal that many researchers strive to achieve through a variety of methods, including balancing parameter number with the output quality. Mascarenhas et al. [198] compared the image classification accuracy of two popular networks, VGG-19 and ResNet-50, observing 97.07% and 97.33% accuracy respectively. Although similar in terms of output quality, VGG-19 possesses 144 million parameters [199], while ResNet only possesses 23 million parameters [200]. This indicates that the network architecture, including number of layers and parameters, is an important factor in minimising the computational cost while maintaining output quality.

A notable challenge to processing the imaging data from CT and other modalities is its volumetric or three-dimensional nature, as CT volumes comprise of a number of individual two-dimensional slices that are “stacked” to form a three-dimensional volume. However, common implementations of neural networks in computer vision tasks take two-dimensional inputs in the form of images or pixel matrices and process them using 2D-covolution operations within the network. This introduces a problem: the relationship between individual slices (across the z-direction) is not preserved, leading to inconsistency in three-dimensional features. Ideally a network could take a three-dimensional input, but this has been observed to significantly increase the computational memory and time required [201]. Variations of input and layer dimensionality have been explored in recent research, focussing on 2D, 2.5D, and 3D. 2.5D methods aim to encourage inter-slice feature preservation by incorporating more than a single slice as input, either through the addition of adjacent slices, or a cross-section encompassing the transverse, coronal, and sagittal planes. A study by Ziabari et al. [202] compared the effectiveness of three dimensionalities (2D, 2.5D, and 3D) of deep learning model-based iterative reconstruction (DL-MBIR) for CT images. Despite improved image fidelity, they observed that reconstruction times for the three methods increased with dimensionality: 7.4 s for the 2D DL-MBIR, 7.7 s for the 2.5D, and 119.4 s for the 3D approach. While 2.5D methods benefit from lower processing time than 3D methods and better performance than 2D methods, the balance between computational demand and quality of output is an ever-present issue.

### Overfitting and the impact of data availability

**Fig. 12:**
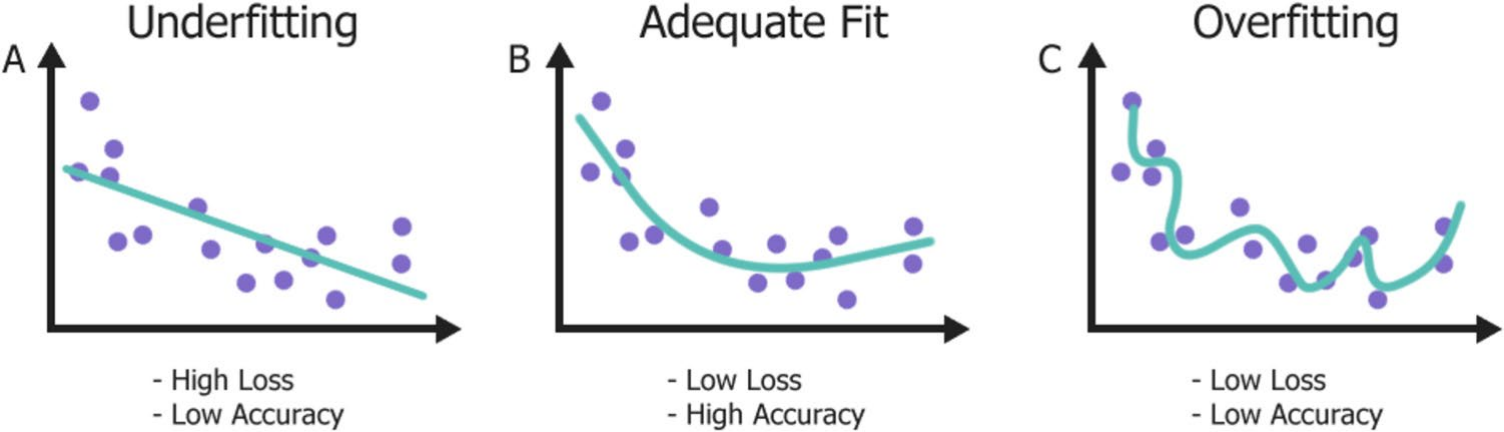
Visualisation of a linear regression model training curves for underfitting (**A**), adequate fit (**B**), and overfitting (**C**)

Traditional supervised learning models require a large amount of labelled data, however, this requires substantial time, expertise, and effort. In addition, some studies are limited by relatively small, specific sample sizes sourced from a single clinical centre, which represents only a narrow portion of the total population. This highly specific training data can lead to network overfitting and a failure to produce adequate results when introduced to unseen datasets. With an insufficiently sized dataset, the network attempts to learn each individual datapoint, neglecting to capture the general trend and overfitting to the data, as shown in Fig. 12. It is statistically important to include a large and diverse sample dataset to adequately represent the population and improve network generalisation [203].

Certain medical imaging modalities, such as CT, require the subject to undergo some level of radiation exposure to produce images. Consequently, to minimise potential harm to patients from unnecessary imaging, data is often sourced from pre-existing databases. Alongside this, medical imaging datasets are not always available in the conditions necessary for the proposed supervised deep learning methodologies, such as including image pairs or accurate labels [204].

Semi-supervised learning overcomes this by including a small portion of labelled examples in an unlabelled dataset. Zhou et al. [205] notes that although the samples do not possess labels, they can be sourced from the same data distribution as the labelled samples. However, this process depends on forming some assumptions about the data distribution. The main assumptions include: the smoothness assumption, the cluster assumption, and the manifold assumption [206]. These allow the underlying data distribution of the unlabelled samples to be joined with the labels. Clustering, for example, assumes that data samples which lie within the same groups (“clusters”) likely belong to the same class. In some situations, using unlabelled data may inhibit the learning performance of a network. As discussed in Li et al. [207], the “safeness” of semi-supervised learning can be dependent on data quality, model uncertainty, and measure diversity.

Another strategy that is often implemented in the literature to avoid overfitting and improve generalisation is data augmentation. For imaging, augmentation may involve supplementing the dataset with new images sourced from existing ones by manipulation. This can be achieved through geometric techniques such as rotation, translation, mirroring, and shearing, or through non-geometric techniques like noise, crop-and-rescale, and colour [208]. Several methods for data augmentation also utilise smaller regions of the input image as “patches”, which can be combined together to create composite images using techniques like random image cropping and patching (RICAP) [209].

Applying regularisation, which penalises neuron weights within the network, also aids in preventing overfitting [210]. A simple technique to incorporate is dropout, which excludes a subset of random neurons each iteration, discouraging dependence [211]. Two common types of regularisation are L1 and L2 regularisation, both of which can be incorporated into the network loss function. Also termed “lasso”, L1 regularisation penalises based on the sum of the absolute value of the weights, whereas L2 regularisation, or “ridge regression”, penalises based on the square of the weights. Both types aim to reduce the weights towards zero, however, L2 regularisation is asymptotic [179]. Other techniques include batch normalisation [212], learning rate decay [213], and reducing model complexity [214].

Incorporating a validation dataset during network training also reduces overfitting. The total dataset collected for network input is often divided into three categories: training, validation, and testing. The training dataset accounts for the highest percentage of the total data, with significantly smaller portions for validation and testing (e.g., 70% training, 15% validation, 15% testing). Samples in the training dataset are used to learn the network parameters required for a desirable output, while the testing dataset is isolated until training is completed to evaluate the performance on new data.[215] The validation dataset is also independent from the training dataset, but functions as an ongoing assessment of the performance during training, allowing for hyperparameters such as number of network layers to be tuned. This ensures that the network generalises sufficiently, avoiding the problem of overfitting.[216] In addition, if a validation dataset is included, overfitting can be avoiding by terminating training when the validation loss stops improving (called “early stopping”) [217].

Alternatively, cross-validation can be used to assess the ability to generalise on unseen data. This method separates the whole dataset into a number of subsets, with one subset held-out for validation or testing. It differs from conventional hold-out (the “train-validation-test split”) through the “cross” mechanism, which changes the test subset each iteration and averages the results [218]. Exhaustive cross-validation methods, like leave one out cross-validation (LOOCV), utilise all possible combinations of subset division. LOOCV is computationally intensive, as the model is trained on n-1 samples and tested on one sample, iterating over the total number of samples. K-fold cross-validation is a non-exhaustive method that reduces the computational load by dividing the dataset into k number of subsets or “folds”, with each subset containing multiple individual samples. For k iterations, a different subset is held-out for validation, such that each sample within is used only once for that purpose before the average is calculated. LOOCV is a more comprehensive, low bias approach due to testing against a single data sample, however, this can also result in higher variance if the sample is an outlier [219].

### Clinical implementation

Publications evaluating the efficacy of deep learning methodologies in medicine demonstrate the technical integrity of their proposed methods, which is only the initial stage involved in clinical implementation. To avoid overestimation of performance, methodology verification requires not only an evaluation of performance metrics, but also its effect on patient outcome through techniques such as clinical trials. As such, many articles published in the field of deep learning for medical imaging require further assessment beyond the initial study before clinical implementation [220]. Further, systematic comparison or meta-analysis between methods relies on multiple similar studies which address the same problem, ideally measuring the same outcomes [221].

Some applications of deep learning in medical imaging are extremely specific, as is the case with the study on aortic valve segmentation via nnU-Net published by Li et al. [183], and as such there are few publications which have a meaningful and direct comparison to their work. This can also be observed in Tables 2 and 3 of this review, as some of the publications do not report the same metrics as other studies. For example, Schaefferkoetter et al.[176] evaluated the effectiveness of using a deep learning network to perform deformable registration of motion-affected PET-CT images through predominantly qualitative assessment. They note that calculating evaluation metrics like the MSE of the deformation vector field (DVF) is not applicable in their assessment, as they do not necessarily correlate to accuracy in the reconstructed PET activity values. The use of these highly specific studies in meta-analysis is limited by the ability to adequately compare results with similar publications systematically.

Integrating AI into healthcare also requires regulatory considerations, ensuring that it is developed and utilised in a responsible and ethical way to benefit everyone. Regulatory groups, such as the Food and Drug Administration (FDA) in the USA and the European Medicines Agency (EMA), have identified a need for guidelines to objectively assess and support the clinical application of AI in healthcare [222, 223]. A review by Joshi et al. [224] examines the characteristics and approval pathway of the 691 FDA-approved AI- and ML-enabled medical devices, as of 2023 [225]. Interestingly, the most prominent medical speciality present in the approved cohort of devices belong to radiology, supported by the vast quantity of research concerned with the improvement of medical imaging with AI. They also observed that a majority of FDA approvals occurred through the 510(k)-clearance pathway, which omits the requirement of exhaustive clinical trials through evidence of substantial equivalence. Significantly, only approximately 3% of the approved devices disclosed the use of clinical trials, which are primarily survey adults within the US. This points out two issues prevalent in deep learning applications: the absence of in-situ evidence through clinical trial, and the use of narrow and homogenous dataset demographic. To overcome this, a framework for the approval and implementation of “software as a medical device” (SaMD) was developed by the International Medical Device Regulators Forum (IMDRF), which includes medical device regulatory bodies from across the globe. It seeks to standardise definitions, risk categorisation, quality management, and evaluation in a clinical setting for cases of SaMD [226]. The Australian Government has similarly determined that AI intended for use as a medical device qualifies under the Australian Register of Therapeutic Goods (ARTG), stating that “clinical and technical evidence must demonstrate the safety, reliability and performance of the product using … AI … to the same standard as other medical devices” [227]. With further understanding and experience, implementation and quality assurance processes can be established for various deep learning applications in clinical settings broadly [220].

#### Examples of current clinical implementation

Although challenges in the implementation of deep learning applications in CT imaging persist, a number of both commercially and non-commercially developed software products have been made available. These software packages are often pretrained on vast amounts of data before deployment, freezing training so that the neural network within does not continue to learn [228].

The objective of deep learning reconstruction (DLR) methodology is to overcome the limitations of the traditional filtered backprojection (FBP) reconstruction approach by performing neural network-assisted denoising. This is especially useful for low-dose CT imaging, which reduces dose to the patient but suffers from increased image noise and artefacts [229]. The GE Healthcare software, TrueFidelity [230], was the first deep learning-based image reconstruction algorithm that became commercially available in 2019. TrueFidelity is supplied as a pretrained deep neural network, demonstrating superior texture preservation, reduced noise and higher spatial resolution than both FBP and adaptive statistical iterative reconstruction [231, 232]. A competing vendor, Canon Medical Systems, have also introduced two DLR algorithms for the suppression of noise in MRI images: advanced intelligent clear-IQ engine (AiCE)[233] and precise IQ engine (PIQE) [234]. The two systems are distinct in task, however, as AiQE was developed with a focus on image denoising and PIQE was developed for super-resolution. More recently, Philips has presented their own DLR approach, Precise Image [235], for low-dose CT reconstruction with inbuilt noise and artefact reduction.

In 2020, Siemens Healthineers introduced two new AI-based assistants to their existing auto-contouring workflow system, AI-Rad Companion (AIRC) [236]. The family of assistants encompass five applications: brain MRI, chest CT, chest x-ray, organs RT, and prostate MR. The AIRC Brain MR for Morphometry Analysis assistant automatically performs segmentation of MRI brain images, supporting measurement of grey and white matter, and cerebrospinal fluid for brain volumetry. Similarly, the Chest CT provides automatic segmentation of the heart, lung lobes, aorta, and vertebra, as well as lung lesion and calcium-content detection.

Due to the commercial nature of vendor-supplied AI- and deep learning-based software products, much of the internal processes are deliberately obscured, reinforcing the “black box” stereotype. Specific model details, such as architecture, hyperparameters, and datasets, are often concealed in an effort to protect competitors from infringing on the intellectual property rights of the vendor. The ability to understand the logic behind the decisions made by the model, or interpretability, assists with reliability through establishing trust in the decision framework. Without an understanding of the inner structure and insight into the machine decision, practitioners are less likely to incorporate deep learning approaches into clinical use [237, 238].

To support the selection and implementation of AI software, an online database for products available on the European market with CE certification has been compiled, currently featuring 224 examples of AI-based software for radiology [239]. The Health AI Register aims to independently and transparently present software choices that are appropriate for potential users in radiology, using vendor-supplied specifications like modality, task, deployment, pricing, and regulatory information. In their associated 2020 publication, van Leeuwen et al. [240] note that, although all highlighted AI software products are CE certified, 36% of them included peer-reviewed evidence, with only 18% demonstrating potential clinical impact.

## Conclusion

The introduction of deep learning methodology and artificial neural networks has allowed for tremendous growth in meeting the needs and paving the way for the future in medical image processing. This review outlined the nature of deep neural networks, including basic principles, common types, network structure, and challenges, as well as a specific discussion of deep learning applications in CT. Five areas that are central to the application of deep learning to CT image processing have been reviewed in detail: denoising, super-resolution, image generation, registration, and segmentation. Selected studies in these areas have been provided to showcase recent advancements in this rapidly evolving field.
